# Modulating the Electrical and Mechanical Microenvironment to Guide Neuronal Stem Cell Differentiation

**DOI:** 10.1002/advs.202002112

**Published:** 2021-02-18

**Authors:** Byeongtaek Oh, Yu‐Wei Wu, Vishal Swaminathan, Vivek Lam, Jun Ding, Paul M. George

**Affiliations:** ^1^ Department of Neurology and Neurological Sciences Stanford University School of Medicine Stanford CA 94305 USA; ^2^ Department of Neurosurgery Stanford University School of Medicine Stanford CA 94305 USA; ^3^ Institute of Molecular Biology Academia Sinica Taiwan

**Keywords:** ciliary neurotrophic factor, conductive polymers, electrical stimulation, electrophysiology, graphene, cell scaffolds, stem cells

## Abstract

The application of induced pluripotent stem cells (iPSCs) in disease modeling and regenerative medicine can be limited by the prolonged times required for functional human neuronal differentiation and traditional 2D culture techniques. Here, a conductive graphene scaffold (CGS) to modulate mechanical and electrical signals to promote human iPSC‐derived neurons is presented. The soft CGS with cortex‐like stiffness (≈3 kPa) and electrical stimulation (±800 mV/100 Hz for 1 h) incurs a fivefold improvement in the rate (14d) of generating iPSC‐derived neurons over some traditional protocols, with an increase in mature cellular markers and electrophysiological characteristics. Consistent with other culture conditions, it is found that the pro‐neurogenic effects of mechanical and electrical stimuli rely on RhoA/ROCK signaling and de novo ciliary neurotrophic factor (CNTF) production respectively. Thus, the CGS system creates a combined physical and continuously modifiable, electrical niche to efficiently and quickly generate iPSC‐derived neurons.

## Graphical abstract

Induced pluripotent stem cells are exciting cells for understanding development and in regenerative medicine. Stem cells respond to their environment to perform their functions and mature. A newly designed conductive scaffold is capable of shaping the mechanical and electrical environment. Utilizing this conductive polymer platform, enhanced stem cell neuronal maturation of the stem cells and important pathways can be identified.

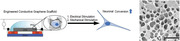

## 1 Introduction

Neurologic conditions such as epilepsy, autism, or Alzheimer's disease cause major morbidity and mortality in children and adults.^[^ ^1^ ^]^ However, endeavors to investigate neuronal activity of the cerebral cortex in health and disease have been hindered by the availability of model systems.^[^ ^2^, ^3^ ^]^ Because induced pluripotent stem cells (iPSCs) are derived from an individual's own cells, they provide an exciting opportunity to create more effective model systems. While efficient strategies using inhibitor cocktails of SMAD and Wnt signaling have been established to promote differentiation of human iPSCs or embryonic stem cells into neural precursor cells (NPCs) rapidly, further maturation into functional neurons in vitro is a lengthy process and has largely lacked the mechanical and electrical cues seen during development.^[^ ^1^, ^4^, ^5^, ^6^, ^7^ ^]^ Additionally, current techniques to develop post‐mitotic cortical neurons such as exposure to trophic factors or neurogenin 2 regulation may not fully recapitulate the myriad of pathways that naturally trigger differentiation.^[^ ^8^, ^9^ ^]^ Bioengineered scaffolds offer a unique platform to begin to address these limitations.^[^ ^10^ ^]^

During neural development, a combination of chemical, mechanical and electrical signals guide stem cell fate.^[^ ^11^, ^12^, ^13^, ^14^ ^]^ To date, inert polymers such as soft hydrogels whose interactions rely on the inherent polymer properties and cannot be continuously modulated have largely focused on the mechanical properties to interact with these neural precursor cells.^[^ ^15^, ^16^, ^17^ ^]^ Cells are able to transduce mechanical perturbation via a signaling cascade involving downstream effectors such as yes‐associated protein (YAP), transcriptional coactivator with PDZ binding motif (TAZ), and small worm phenotype and mothers against decapentaplefic proteins (SMAD) to maintain stem cell pluripotency.^[^ ^18^, ^19^, ^20^ ^]^ Electrical stimulation has also been found to play an important role in developing neurons and plays a role in epigenetic reprogramming and gene signaling.^[^ ^21^, ^22^ ^]^ This electrical activity during early neuronal development suggests an essential role in cell differentiation and maintenance of phenotype. In embryonic cells, for example, voltage‐gated sodium and calcium channels generating electrical signals are critical for the development of neuronal precursors and differentiating neurons.^[^ ^23^ ^]^ Additionally, in mice hippocampal precursor cells, excitation of cultured cells with voltage‐gated calcium channels represses glial fate genes and induces expression of neural fate genes.^[^ ^12^ ^]^ Owing to the importance of activity‐dependence in early neural development and physiological maintenance, electrical stimulation is also known to be important in developing neurons and can be expected to play a role in determining cell fate. Indeed, while previous studies have demonstrated the enhancement of neuronal differentiation by the application of electrical fields,^[^ ^24^ ^]^ only a limited set used a combination with mechanical influences. We asked whether applying both mechanical forces and electrical stimulation may affect the speed in which pro‐neuronal differentiation occurs in culture. Conductive polymers provide a novel platform to interact with stem cells even after seeding and provide a microenvironment with tunable electrical stimulation.

Here, we explore utilizing a new conductive graphene scaffold (CGS) to enhance direct‐differentiation of iPSCs to a human cortical neuronal fate. By altering mechanical properties of CGS through a new method of nanoconfinement of carbon nanofibers (CNFs) utilizing chemical reduction of graphene oxide (GO). We were able to alter the stiffness of the scaffold by controlling the ratio of CNF to graphene oxide. Moreover, an exposure to electrical stimulation provided a rapid procedure to obtain iPSC‐derived neurons with more mature molecular signature and electrophysiological characteristics. To evaluate the underlying molecular mechanism for such effect, we also performed augmentation or reduction experiments and found Ras homolog family member A (RhoA) and ciliary neurotrophic factor (CNTF) were important for CGS‐mediated iPSC conversion to neurons.

## 2 Results

### 2.1 3D CGS Promotes Neuronal Conversion of iPSCs

**Figure 1:**
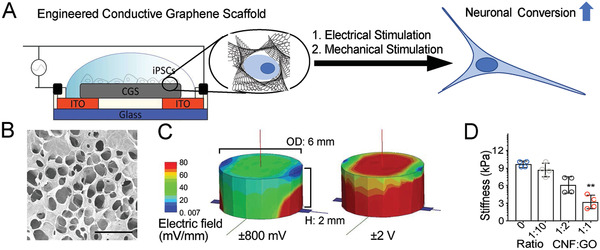
3D CGS for human iPSC culture. A) Schematic illustration of electrical stimulation chamber, showing iPSCs on a conductive graphene scaffold (CGS) to which a specific voltage and frequency are applied. B) SEM image of the surface morphology of CGS. Scale bar indicates 100 µm. C) Computational simulation of applied electric field spatial distribution on the surface of soft CGS (left, ±800 mV; right, ±2 V). D) Bar graph showing stiffness of CGS (ranging from ≈12 to ≈3 kPa as CNF proportion is increased). D) Analyzed using a one‐way ANOVA, followed by Tukey's HSD post hoc test with ^*^ *p* < 0.05 and ^**^ *p* < 0.01 compared to 0 ratio CGS. Values represent the mean of independent experiments (*n* = 4); error bars, SD.

Electrical activity plays an important role in modifying neural activity^[^ ^25^, ^26^ ^]^ along with other environmental factors modulating stem cell reorganization, migration, proliferation, and differentiation.^[^ ^27^, ^28^, ^29^, ^30^ ^]^ To harness these effects, we prepared a unique 3D macroporous and mechanically soft CGS capable of electrical stimulation using chemical reduction of graphene oxide (rGO) combined with CNFs (**Figure** **1**A–C; Figure. S1A–D, Supporting Information). In contrast, a 2D CGS demonstrated a less porous structure (Figure S1E,F, Supporting Information). An additional benefit of the CGS platform is that it is made via a straightforward process using readily available products. Modeling the distribution and gradient of the electrical field across the CGS (Figure 1C) found a relatively uniform electric field (±800 mV; 100 Hz). By simply varying the ratio of CNFs, we were able to alter the stiffness of the scaffold, with higher concentrations of CNFs resulting in softer scaffolds (1:1 of CNF:GO ≈3 kPa) (Figure 1D). The conductivity of the CGS did not vary across different stiffness with the stiff CGS containing the least amount of CNF and soft CGS containing the highest concentration of CNF (Figure S1A, Supporting Information). On the cross‐sectional plane, the CGS morphologically was aligned and rGO was intertwined with CNFs (Figure S1B, Supporting Information).

Mechanotransduction is a key parameter for cell migration, proliferation, and differentiation.^[^ ^31^, ^32^, ^33^, ^34^ ^]^ It has been established that the Hippo/YAP pathway of pluripotent stem cells on different stiffness of polymeric hydrogels is a regulator for pluripotent stem cell differentiation into neurons.^[^ ^19^ ^]^ Traditional iPSC culture techniques use dual SMAD inhibitors, resulting in promoting neuronal conversion.^[^ ^1^ ^]^ Moreover, it has been recently observed that RhoA, a cytoskeletal dynamics regulator, is a main effector on neuronal differentiation of murine pluripotent stem cells via SMAD downregulation.^[^ ^35^ ^]^ Because of this, we evaluated if changes in stiffness of the CGS increased the rate of neuronal differentiation on our CGS and if this was related to changes in the YAP/p‐SMAD pathway through RhoA downregulation in human iPSCs.

**Figure 2:**
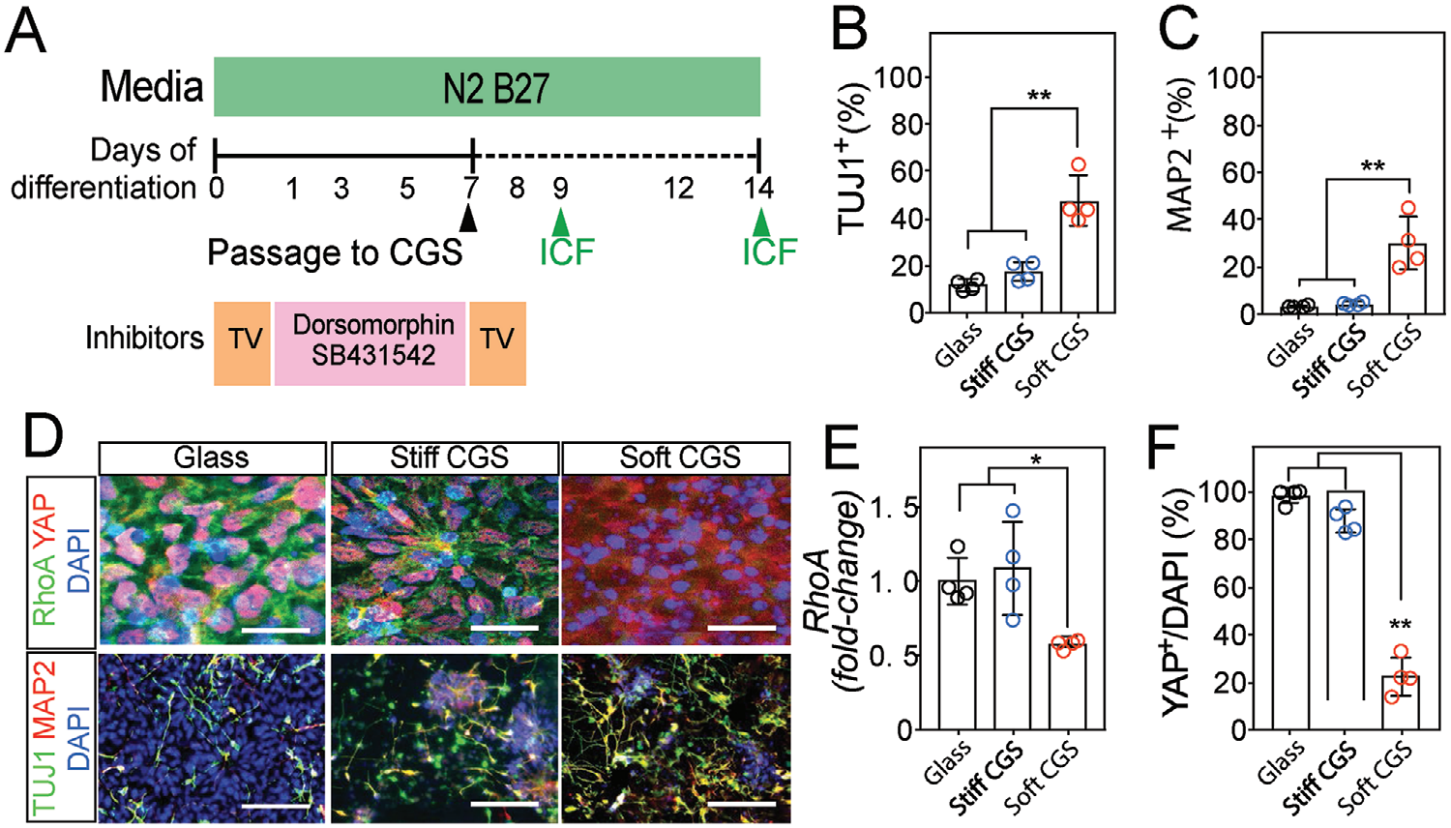
Soft CGS promotes RhoA downregulation and neuronal conversion. A) Differentiation scheme for CGS‐based neuronal induction protocol. Human iPSCs initially preconditioned with N2B27 media supplemented with dual SMAD inhibition (Dorsomorphin and SB431542) for 7d were passaged onto CGSs. After passaging, the cells were maintained with N2B27 media without dual SMAD inhibitor. ICF, immunocytofluorescence (green arrow). Bar plots showing percentages of B) TUJ1^+^ and C) MAP2^+^ at 7d on CGSs of varying elasticity. D) Representative immunocytofluorescence analysis of RhoA (green)/YAP (red) and TUJ1 (green)/MAP2 (red) in iPSCs cultured on glass, stiff, and soft CGS. Cell nuclei were counterstained with DAPI (blue). Scale bars indicate 25 for RhoA/YAP and 50 µm for TUJ1/MAP2, respectively. E) qRT‐PCR analysis of *Rhoa*, an intracellular signal mediator, in iPSCs cultured on glass, stiff or soft CGS. Expression levels are normalized to GAPDH. F) Bar plot showing rigidity‐dependent nuclear co‐localization of YAP in iPSCs cultured for 2d on different substrates. B,C,E,F) Analyzed using a one‐way ANOVA, followed by Tukey's HSD post hoc test with ^*^ *p* < 0.05 and ^**^ *p* < 0.01. Values represent the mean of independent experiments (*n* = 4); error bars, SD.

Preconditioning of iPSCs under N2B27 media supplemented with dual SMAD inhibitors (Dorsomorphin and SB431542) generated PAX6^+^/Nestin^+^ neural precursor cells (**Figure** **2**A and Figure S2A–D, Supporting Information). To determine whether these precursor cells undergo selective differentiation on stiffer CGSs, we tested CGSs with varying elasticity (≈3 to 12 kPa). The soft CGS substrate was more efficient at increasing expression of early and mature neuronal markers (TUJ1^+^ and MAP2^+^) than the stiff CGS or glass substrates (Figure 2B–D). Without the 3D structure, the 2D CGS produced less early and mature neuronal markers (Figure S2E,F, Supporting Information).

Next, we examined if the RhoA and YAP/p‐SMAD pathways were involved with these changes as observed in previous studies.^[^ ^19^, ^35^ ^]^ The expression level of *Rhoa* from iPSCs on both glass (control) and stiff CGS were similar (Figure 2E). Notably, iPSCs on soft CGS had decreased *Rhoa* transcription. Given that *Rhoa*‐dependent F‐actin polymerization occurs at the cell periphery,^[^ ^35^ ^]^ we expected differences between the peripheral stress fibers in cells cultured on substrates of varying elasticity. Quantification of F‐actin in cells of each group estimated the difference in cytoskeletal stress (Figure S2G,H, Supporting Information). Peripheral stress fibers, visualized by staining for F‐actin, were decreased in neural precursor cells cultured on soft CGS compared to the cells on glass and stiff CGS, consistent with the reduction of *Rhoa* activity in the soft CGS.

Immunocytofluorescence analysis revealed that RhoA and YAP/p‐SMAD expression were regulated by substrate stiffness (Figure 2D–F and Figure S2G,I, Supporting Information). A decrease in F‐actin polymerization associated with less *Rhoa* transcription has been linked to reduced transcriptional regulatory activity of YAP and p‐SMAD.^[^ ^19^, ^20^ ^]^ In our experiments, YAP and p‐SMAD are localized in the nucleus on both the glass and stiff CGS, whereas it is mainly excluded from the nucleus on the soft CGS (Figure 2D,F and Figure S2G,I, Supporting Information). Previously, YAP/p‐SMAD have been implicated in signaling pathways elicited by mechanical stimuli.^[^ ^18^, ^19^, ^20^ ^]^ Cell cultures on the soft CGS exhibited efficient p‐SMAD and YAP sequestering with significant decreases in the proportion of cells with co‐localization in the cell nucleus (Figure 2D,F and Figure S2I, Supporting Information).

Taken together, our experiments indicate that the mechanical stimuli conferred by the CGS platform are more efficient at promoting pro‐neuronal markers than the glass substrate alone. Additionally, YAP/p‐SMAD sequestration coincides with RhoA downregulation in rigidity‐dependent, neuronal differentiation of iPSCs (Figure S2J, Supporting Information). We found that decreasing the stiffness of the CGS augmented early neuronal conversion of iPSCs and that RhoA is implicated in this regulation which is consistent with previous results.^[^ ^19^ ^]^ Further experiments are required to demonstrate a causal relationship, but our findings demonstrate the importance of this pathway in the CGS system.

### 2.2 Electrical Stimulation Augments the Generation of iPSC‐Derived Neurons

**Figure 3:**
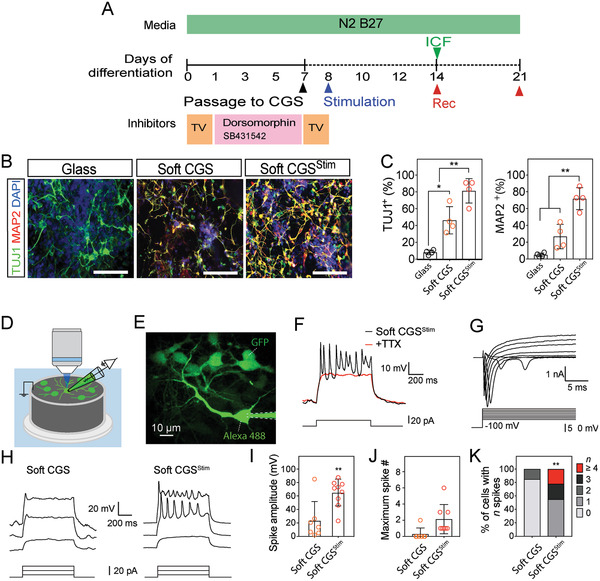
Electrical stimulation increases mature characteristics of iPSC‐derived neurons. A) Schematic of the differentiation procedure. Electrical stimulation: AC, ±800 mV, 100 Hz for 1 h. ICF immunocytofluorescence. Rec, electrophysiological recording. B) Representative images of TUJ1^+^ (green) and MAP2^+^ (red) cells on different conditions. C) Bar plot showing the percentage of cells labeled with TUJ1^+^ or MAP2^+^. D) An illustration of fluorescence‐guided whole‐cell patch‐clamp recording on CGS. E) The morphology of a whole‐cell patch‐clamp recorded and surrounding eGFP‐expressing iPSC‐derived cells by two‐photon imaging. Scale bar indicates 10 µm. F) Traces of multiple action potentials triggered in an iPSC‐derived cell cultured on soft CGS^Stim^ for 14d (black). Tetrodotoxin (TTX) completely prevented the generation of action potentials (red). G) Traces of membrane current in response to step voltage‐clamp from −100 to +10 mV. H) Representative membrane potentials upon step current injections of iPSC‐derived cells cultured 14d on soft CGS and soft CGS^Stim^. I) The summary results of averaged spike amplitude from iPSC‐derived cells without or with electrical stimulation. J,K) Maximum spike number and percentage of cells with indicated firing frequencies at 14d of differentiation on soft CGS without or with electrical stimulation. B) Cell nuclei were counterstained with DAPI (blue). Scale bars indicate 100 µm. C) Analyzed using a one‐way ANOVA, followed by Tukey's HSD post hoc test with ^*^ *p* < 0.05 and ^**^ *p* < 0.01. Values represent the mean of independent experiments (*n* = 4); error bars, SD. I,J) Analyzed using a paired Student's *t*‐test with ^*^ *p* and ^**^ *p* < 0.01 and 0.05, respectively. *N* = 7 and 9 for soft CGS and soft CGS^Stim^ from four batches of independent cell cultures. K) Analyzed using a Fisher exact test with ^**^ *p* < 0.01.

Electrical stimulation is able to alter the transcriptome of stem cells.^[^ ^22^, ^36^, ^37^ ^]^ Due to the lack of conductivity in traditional culture systems and material limitations (i.e., inert hydrogel or scaffold), the combination of electrical and mechanical cues on guiding iPSC differentiation remains largely unexplored. Utilizing the properties of our CGS platform, we simultaneously applied mechanical and electrical stimulation to iPSCs to determine the effect on neuronal differentiation. Previous studies demonstrated that a single 1 h period of electrical stimulation results in sustained alteration of progenitor cell gene expression.^[^ ^22^, ^38^ ^]^ Utilizing these parameters for duration of stimulation, the voltage for electrical stimulation of the iPSCs was optimized by assessing neuronal differentiation from iPSCs for CGS stiffness (elasticity ranging from 3 to 12 kPa) and voltage (applied voltages ranging ±100 mV to ±3 V) (**Figure** **3**A; Figure S3 and Table S1, Supporting Information). Exposure to ±800 mV at 100 Hz for 1 h on the soft CGS (≈3 kPa) significantly increased the generation of TUJ1^+^ neurons without adverse cytotoxicity after 7d culture on the CGSs (Figure S3A–D and Table S1, Supporting Information). These studies show that stimulation with a voltage of greater than 1000 mV increases cell death (Figure S3A,B, Supporting Information) while reducing the percentage of cells expressing immature neuronal markers (Figure S3C, Supporting Information). To investigate the impact of the frequency of stimulation on the cells, we evaluated immature and more mature neuronal markers (TUJ1 and MAP2, respectively) across several frequencies (i.e., direct current (DC), alternating current (AC) 10, 50, and 100 Hz). We found that stimulation with AC frequencies promotes TUJ1‐positive neurons (Figure S3E, Supporting Information). Furthermore, the higher frequency stimulation (100 Hz) caused an increase in more mature MAP‐2 positive neurons. Based upon these results, we chose the optimum stimulation parameters of ±800 mV at 100 Hz in the subsequent studies to better understand how the pro‐neuronal effect may be conferred through the use of the mechanically and electrically interactive CGS system. With these stimulation parameters, the expression of TUJ1 and MAP2 were highly increased by electrical stimulation relative to unstimulated groups (Figure 3B,C and Figure S3F, Supporting Information).

To confirm that iPSC‐derived neurons using soft CGS^Stim^ differentiate into electrophysiologically active neurons, we used a fluorescence‐guided approach to perform whole‐cell patch‐clamp recordings (Figure 3D) and confirm the morphology of the differentiated neurons with two‐photon imaging (Figure 3E). After 7d of culture on the CGS, a small spike‐let was observed from iPSC‐derived neurons in the soft CGS^Stim^ condition, whereas no distinct spike‐let was found in soft CGS without an exposure to electrical stimulation (Figure S3G, Supporting Information). After 14d of culture on the scaffolds, full action potentials were repetitively induced in response to step current injection. The action potentials were sensitive to the blockage of voltage‐gated sodium channels by tetrodotoxin (TTX, 1 × 10^−6^ m; Figure 3F). Typical biphasic inward‐ and outward‐currents from voltage‐gated sodium and potassium channels were also observed (Figure 3G), suggesting the iPSC‐derived neurons are electrophysiologically functional on the soft CGS^Stim^. Furthermore, the average spike amplitude was significantly larger with soft CGS^Stim^ (*p* < 0.01 compared with soft CGS without electrical stimulation, Figure 3H–K). Utilizing the soft CGS^Stim^, 100% of the iPSC‐derived neurons were capable of firing, with 20% neurons showed more mature firing patterns (Figure 3J,K). This time frame is comparable or faster than that achieved by some previous pluripotent stem cell‐based neuronal differentiation methods and does not require specialized mechanobiology or genome transfection (Table S2, Supporting Information).

To determine how the CGS platform compared to a standard small molecule technique, immunofluorescent and electrophysiological characterization of iPSC‐derived neurons using a standard technique for neural differentiation media was performed.^[^ ^5^ ^]^ The electrophysiological and neural markers were not as mature as those seen with the soft CGS with electrical stimulation at 21d (Figure S4, Supporting Information).

### 2.3 Electrical Stimulation Promotes Neurotrophic Factor Signaling

**Figure 4:**
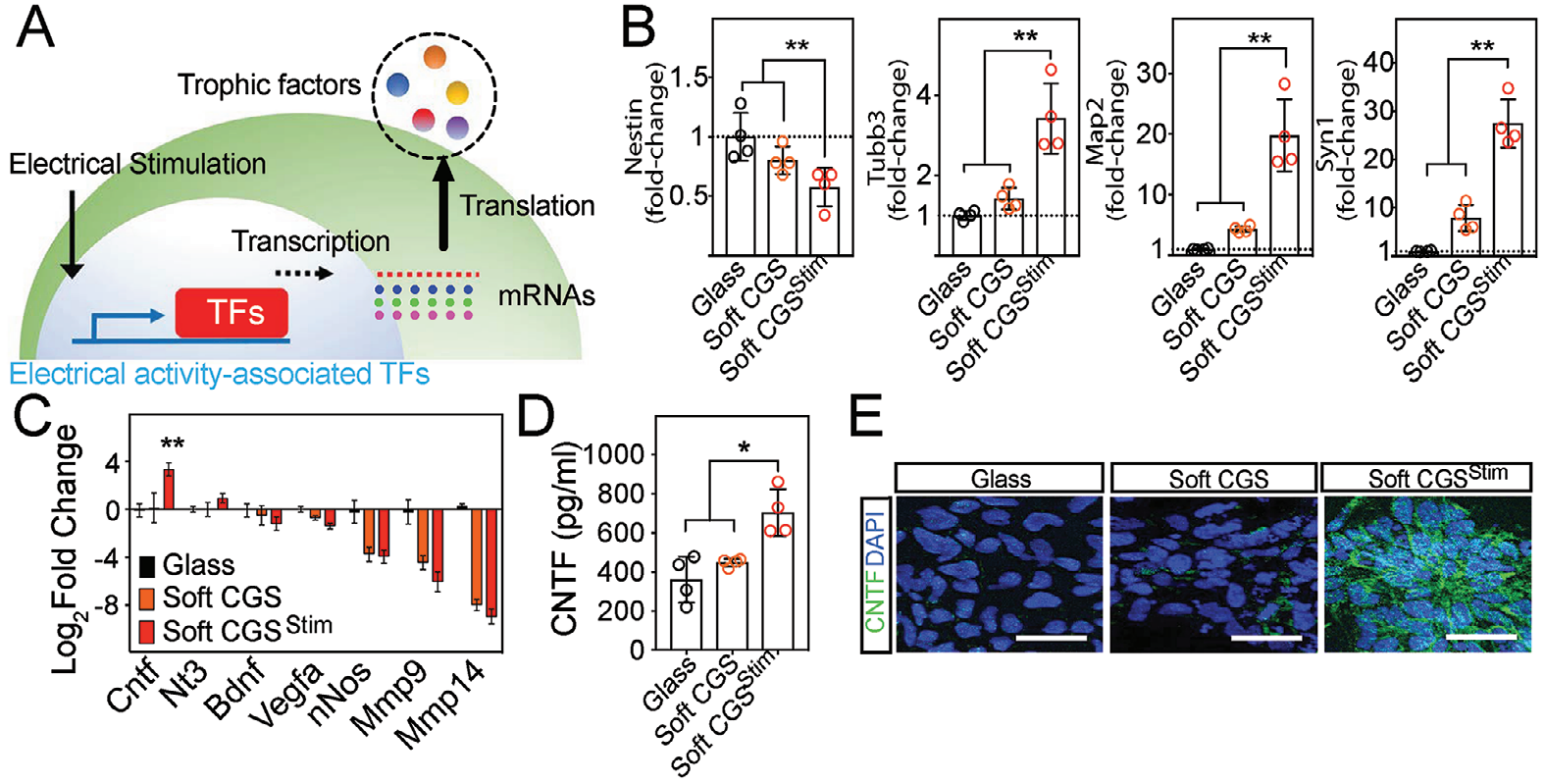
Electrical stimulation‐associated CNTF expression exerts a marked impact on neuronal conversion. A) Illustration of pathway manipulation with electrical stimulation. Electrical stimulation increases transcription factors, which will be translated as trophic factors. B) qRT‐PCR analysis of neuronal markers: *Nestin*, neuroectodermal stem cell marker; *Tubb3*, early neuronal marker; *Map2* and *Syn1*, matured‐neuronal marker, at 7d on glass, soft CGS, and soft CGS^Stim^. C) The expression levels of neurotrophic factor genes including *Mmp14, Mmp9, nNos*, *Vegfa*, *Bdnf*, *Nt3*, and *Cntf* at 1d after electrical stimulation. Data are represented as Log_2_ such that positive values indicate upregulation and negative values indicate downregulation relative to cells on glass substrate. D) CNTF in the supernatants of samples with varying substrates was determined by ELISA. E) Immunocytofluorescence analysis for CNTF (green) on control (glass), soft CGS, and soft CGS^Stim^. Cell nuclei were counterstained with DAPI (blue). Scale bars indicate 25 µm. B–D) Analyzed using a one‐way ANOVA, followed by Tukey's HSD post hoc test with ^*^ *p* < 0.05 and ^**^ *p* < 0.01. Values represent the mean of independent experiments (*n* = 4); error bars, SD.

To explore the mechanisms by which an exposure to electrical stimulation promotes neuronal conversion, we evaluated how gene expression of neuronal markers differed between unstimulated and electrically stimulated iPSCs (**Figure** **4**A,B). Gene expression analysis confirmed downregulation of the neuroectodermal stem cell marker *Nestin* and induction of neural markers including *Tubb3, Map2*, and *Syn1* in soft CGS and soft CGS^Stim^. In addition, the exposure to electrical stimulation drastically induced the efficiency of neural markers in stiff CGS^Stim^ (Figure S5A, Supporting Information). This indicates that electrical activity‐associated transcription factors increased, resulting in neuronal differentiation.

Given the induction of neural markers with electrical stimulation, genes of interest were identified from previous work showing transcriptome changes in neural stem cells from electrical stimulation.^[^ ^22^ ^]^ Candidate neurotrophic genes were evaluated with real‐time quantitative reverse transcription polymerase chain reaction (qRT‐PCR). CNTF changed significantly with electrical stimulation on both stiff and soft CGS (Figure 4C and Figure S5b, Supporting Information). Genes such as *Mmp14*, *Mmp9*, *nNos*, *Vegfa*, *Bdnf*, and *Nt3* did not change significantly with electrical stimulation on the CGS. To assess if similar increases were observed in protein production, enzyme‐linked immunosorbent assay (ELISA) results confirmed that CNTF levels were elevated by electrical stimulation independent of substrate stiffness (Figure 4D and Figure S5c, Supporting Information). Additionally, immunocytofluorescence verified the increased expression of the CNTF protein after electrical stimulation of iPSCs on stiff and soft CGS (Figure 4E and Figure S5D, Supporting Information). Immunocytofluorescence analysis to further characterize iPSCs on the scaffolds revealed that after 7d of differentiation (or 14d from iPSCs), the differentiated neurons on soft CGS and soft CGS^Stim^ did not express Nestin; but the majority of cells did express TUJ1 (Figure S5E, Supporting Information). Staining for the proliferation marker, Ki67, revealed more proliferation on glass and stiff CGS compared to the soft CGS, suggesting further maturation of cells on the soft CGS (Figure S5F, Supporting Information). However, glial marker (GFAP) positive cells were not significantly different between groups within this timeframe (Figure S5G, Supporting Information). Additionally, qRT‐PCR quantification of the CNTF release at 5d after stimulation from the NPCs demonstrates that the single time point of electrical stimulation causes sustained increase in CNTF expression for multiple days (Figure S6, Supporting Information).

### 2.4 Altering Combined Pathways Involved in Mechanical and Electrical Stimulation Orchestrates Neuronal Conversion

**Figure 5:**
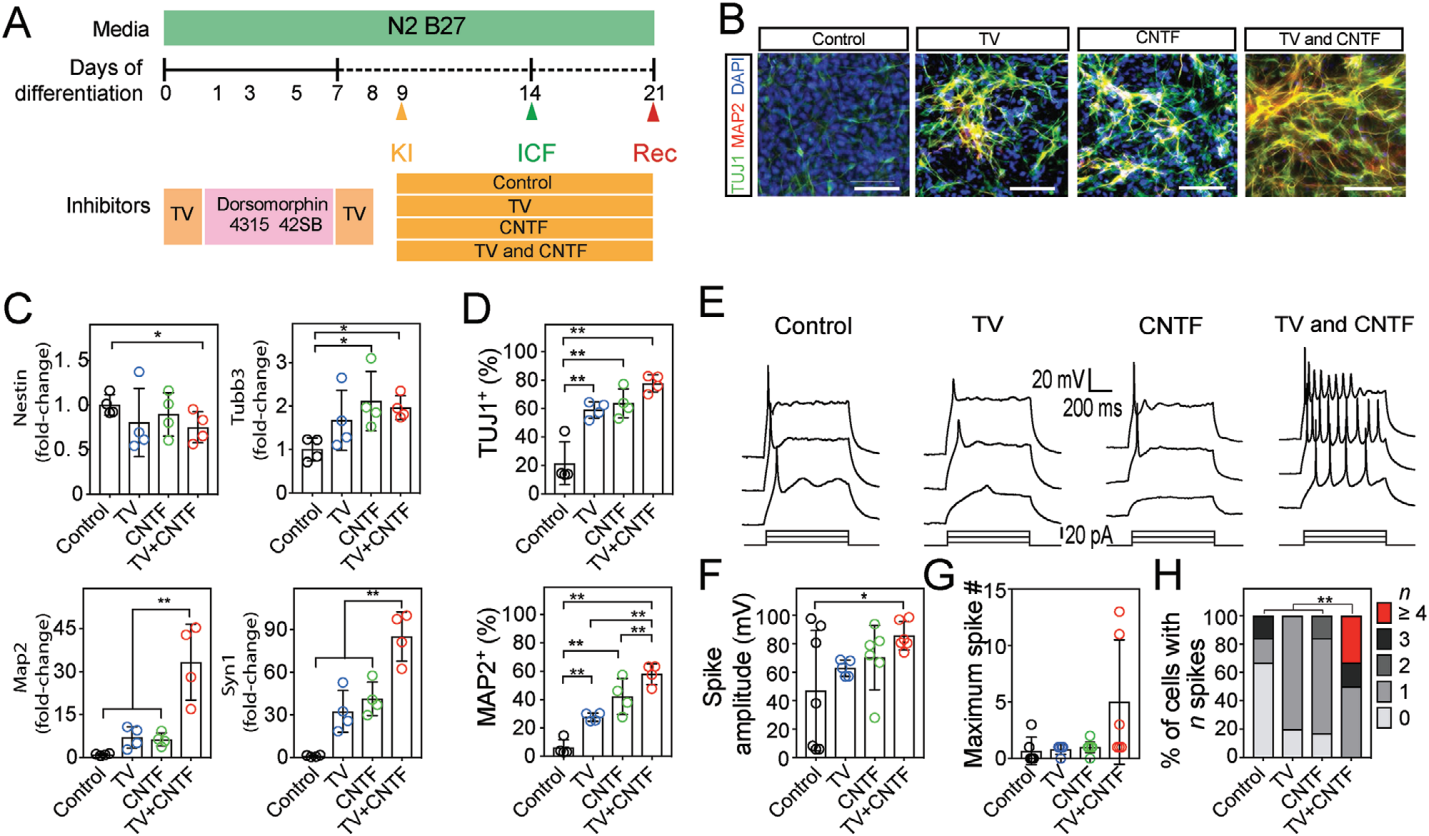
Pathways modulated by mechanical and electrical stimulation orchestrate the emergence of neuronal conversion. A) Schematic demonstrating introduction of factors to mimic important pathways of mechanical and electrical stimulation. B) Representative images of TUJ1^+^ (green) and MAP2^+^ (red) cells on glass substrate (control) in the presence of different treatments (TV (2 × 10^−6^ m), CNTF (1 ng mL^−1^), and TV+CNTF). Cell nuclei were counterstained with DAPI (blue). C) qRT‐PCR analysis of *Nestin*, *Tubb3*, *Map2*, and *Syn1* at 7d after the addition of treatments. D) Bar plot showing the proportion of TUJ1^+^ and MAP2^+^ cells on glass with different conditions. E) Representative traces of membrane potentials in response to step current injection of iPSC‐derived cells after 14d culture on glass substrate after treatment with TV, CNTF, or both (TV+CNTF). F) Bar graph showing the mean spike amplitude from iPSC‐derived cells from different treatments. G,H) Maximum spike number and quantification of percentage of cells with indicated firing frequencies at 14d of differentiation on glass with addition of factors. C,D) Analyzed using a one‐way ANOVA, followed by Tukey's HSD post hoc test with ^**^ *p* < 0.01. Values represent the mean of independent experiments (*n* = 4); error bars, SD. F) Analyzed a one‐way ANOVA, followed by Tukey's HSD post hoc test with ^*^ *p* < 0.05. From left to right, *N* = 6, 5, 6, and 6 from four batches of independent cell cultures. H) Analyzed using a Fisher exact test with ^**^ *p* < 0.01.

The soft CGS^Stim^ combines mechanical and electrical cues and was the most effective stimulation paradigm to generate iPSC‐derived neurons (Figure 3). Compared to published differentiation protocols, the combined mechanical and electrical stimulation is as efficient for neuronal differentiation, if not more so, than those using glass substrate with exogenous factors (Table S2, Supporting Information). Consequently, we postulated that modulating the specific pathways altered by soft CGS^Stim^ as described above and utilizing similar mechanisms as previously described would also influence neuronal differentiation of iPSCs cultured on a traditional glass substrate. To recapitulate changes in the RhoA/Rho‐associated protein kinase (ROCK) expression seen with the soft CGS, we applied the RhoA/ROCK inhibitor, Thiazovivin (TV). To determine if the autocrine feedback from neurotrophic factors modulated by electrical stimulation including VEGFA, BDNF, NT3, and CNTF was critical, we applied exogenous factors to the iPSCs cultures (Figure S7A, B, Supporting Information). It was found that the addition of CNTF plays a critical role in the generation of neurons as compared to others. Then, we examined if the combination of main pathways altered by mechanical (RhoA) and electrical (CNTF) stimulation produced greater conversion to mature neuronal cultures than affecting either pathway in isolation (**Figure** **5**A,B).

iPSCs plated on glass were treated with TV alone, CNTF alone, or both TV and CNTF (TV+CNTF). The similarity between the cells propagated on the glass surface with TV+CNTF treatment and soft CGS^Stim^ is striking. Interestingly, expression of the mature neuronal genes *Map2* and *Syn1* were significantly increased in TV+CNTF culture (Figure 5C). Consistent with this result, seven days after TV+CNTF treatment, the iPSCs‐derived neurons exhibit mature neuronal formation with significant increases in the number of TUJ1 and MAP2 neurons relative to the control glass substrate (Figure 5D).

Next, we tested if the presence of stiffness‐ and electrical‐stimulation associated factors, TV and CNTF, are sufficient to promote the emergence of the neuronal electrophysiological properties in a standard culture system. The presence of TV or CNTF alone slightly increased the averaged action potential amplitudes but did not reach a significance (*p* = 0.57 and 0.67 for TV and CNTF compared with control, respectively, Figure 5E,F). Using combined treatment (TV+CNTF), the iPSC‐derived neurons exhibited multiple action potentials in response to step current injections (Figure 5G). Moreover, the action potential amplitudes were significantly larger (*p* < 0.05 compared with control, Figure 5F). With the combined treatment (TV+CNTF), most of the iPSC‐derived neurons were capable of firing, with ≈30% of neurons showing significantly more mature firing patterns (Figure 5H). These results demonstrate that combination treatment by two different pathways (i.e., mechanical and electrical stimulation) can dramatically improve the efficacy of in vitro iPSC neuronal differentiation.

To more deeply explore whether soft CGS^Stim^ and the combined TV+CNTF promoted earlier conversion of iPSC‐derived neurons as opposed to altering electrophysiological characteristics, we assessed for immature and more mature neuronal markers (Tuj1 and MAP2, respectively) at an earlier time point (day 9 from iPSCs). We found that at the earlier time, more of the iPSC‐derived neurons had mature characteristics suggesting that the new methods promote accelerated maturation of the iPSC‐derived neurons (Figures S8 and S9, Supporting Information).

### 2.5 CNTF Mediates Electrical Stimulation‐Enhanced iPSC Neuronal Differentiation

**Figure 6:**
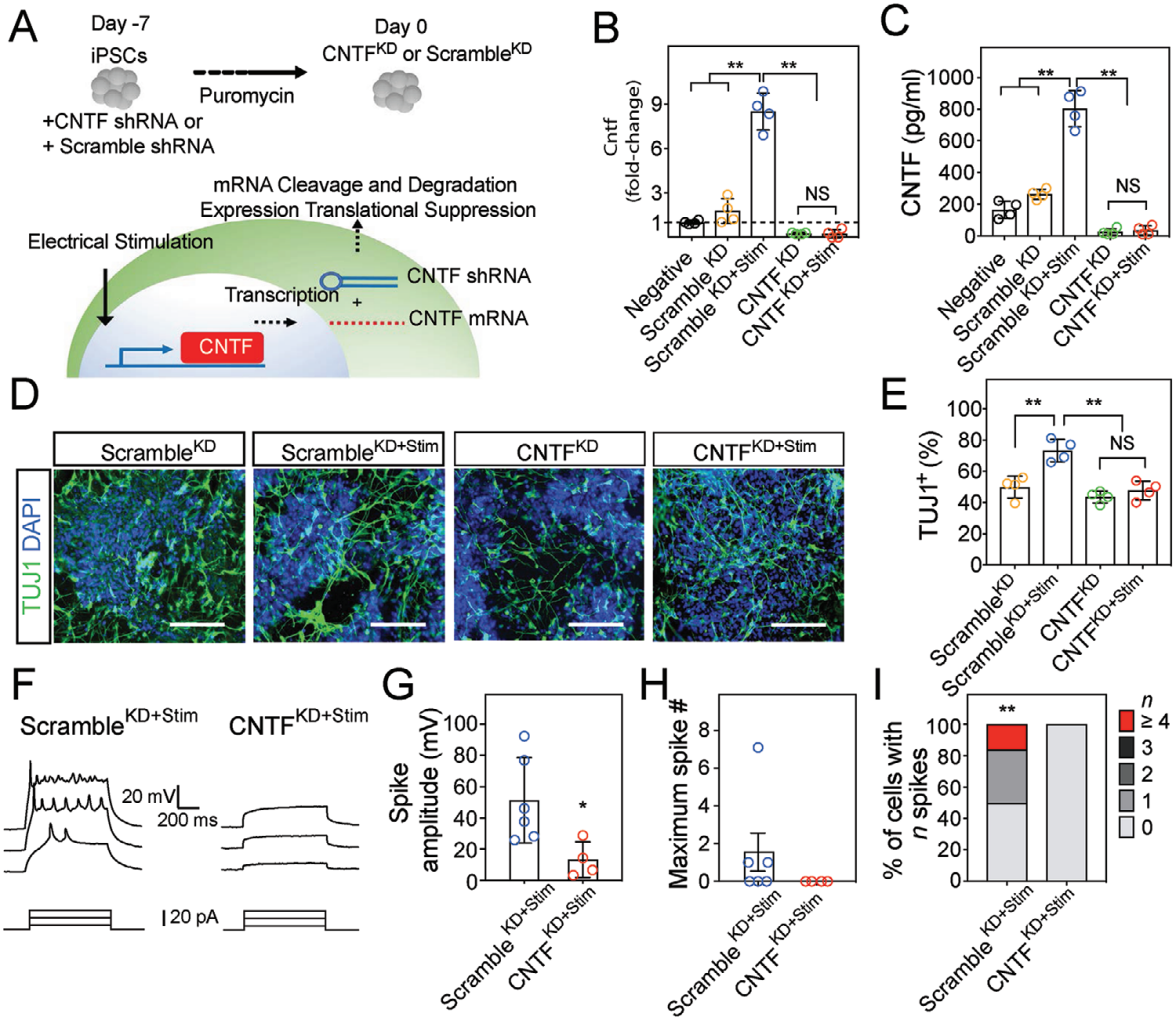
Neuronal conversion of iPSCs on soft CGS^Stim^ was attenuated with Cntf gene knockdown. A) Schematic demonstrating shRNA blocking CNTF mRNA expression. B) qRT‐PCR analysis of *Cntf*. C) CNTF in the supernatants of samples was determined by ELISA. D) Immunocytofluorescence analysis of TUJ1^+^ (green) cells on soft CGS with or without an exposure to the stimulation. Cell nuclei were counterstained with DAPI (blue). Scale bars indicate 100 µm. E) Quantification of TUJ1^+^ cells cultured on soft CGS. iPSCs modified by CNTF^KD^ with electrical stimulation (CNTF^KD+Stim^) did not show a statistical significant difference compared to CNTF^KD^ (without an exposure to the stimulation). F) Representative traces of membrane potentials of iPSC‐derived cells on soft CGS^Stim^ with the control scrambled knockdown (Scramble^KD+Stim^, left) and CNTF knockdown (CNTF^KD+Stim^, right). G) The summary result of averaged spike amplitude of Scramble^KD+Stim^ and CNTF^KD+Stim^ conditions. H,I) Maximum spike number and quantification of percentage of cells with indicated firing frequencies at 14d of differentiation on soft CGS with electrical stimulation. CNTF^KD^ was tested and Scramble^KD^ was utilized as a control group. B,C,E) Analyzed using a one‐way ANOVA, followed by Tukey's HSD post hoc test with ^**^ *p* < 0.01. NS indicates no‐significance between CNTK^KD^ and CNTF^KD+Stim^ (*p* > 0.99, *p* = 0.99, and *p* = 0.77, respectively). Values represent the mean of independent experiments (*n* = 4); error bars, SD. G,H) Analyzed using a paired Student's t‐test with ^*^ *p* < 0.05, respectively. *N* = 6 and 4 for Scramble^KD+Stim^ and CNTF^KD+Stim^, respectively from four batches of independent cell cultures.

To address CNTF's causative role in the rapid appearance of iPSC‐derived neurons after electrical stimulation, *Cntf* expression was reduced by *Cntf*‐shRNA knockdown (KD) (CNTF^KD^) with or without stimulation and compared with controls (scrambled‐shRNA, Scramble^KD^, **Figure** **6**A,B and Figure S10, Supporting Information). Subsequent ELISA studies also reveal that CNTF production decreased in CNTF^KD^ with or without an exposure to electrical stimulation (Figure 6C). iPSCs with scrambled‐shRNA (Scramble^KD^) did not alter the expected increase in TUJ1^+^ cells after electrical stimulation (Figure 6D,E). However, the proportion of TUJ1^+^ cells in both CNTF^KD^ groups (CNTF^KD^ and CNTF^KD+Stim^) were significantly decreased from the scramble plus electrical stimulation group (Scramble^KD+Stim^) and similar to the unstimulated scramble group (Scramble^KD^). These results demonstrate that the enhanced neuronal differentiation of iPSCs seen with electrical stimulation was not observed without CNTF, indicating CNTF as a possible mechanism.

We next investigated whether CNTF is also essential for the iPSC‐derived neurons to express neuronal electrophysiological properties (Figure 6F–I). Indeed, selective knockdown of CNTF with shRNA (CNTF^KD+Stim^) prevented the induction of robust and repetitive induced action potentials upon current injection, whereas in the scrambled shRNA control (Scramble^KD+Stim^) the spiking activity was preserved (Figure 6F,G). In Scramble^KD+Stim^, iPSC‐derived neurons were capable of firing (≈50% of cells), with ≈20% neurons showed more mature firing patterns (Figure 6H,I). However, iPSC‐derived neurons in CNTF^KD+Stim^ were not capable of firing (≈0%) (Figure 6I). These results suggest that CNTF upregulated by an exposure to electrical stimulation is necessary to trigger the differentiation of iPSCs to electrophysiologically active neurons. By blocking CNTF expression, we do not see the rapid differentiation into mature neurons which supports our above conclusions that CNTF is an essential pathway for the pro‐neuronal changes of electrical stimulation. These results further support prior studies which indicate that electrical stimulation can alter gene expression in progenitor cells and are a key mechanism for downstream effects.^[^ ^22^, ^38^, ^39^ ^]^

### 2.6 Characterization of Maturation of the iPSC‐Derived Neurons

**Figure 7:**
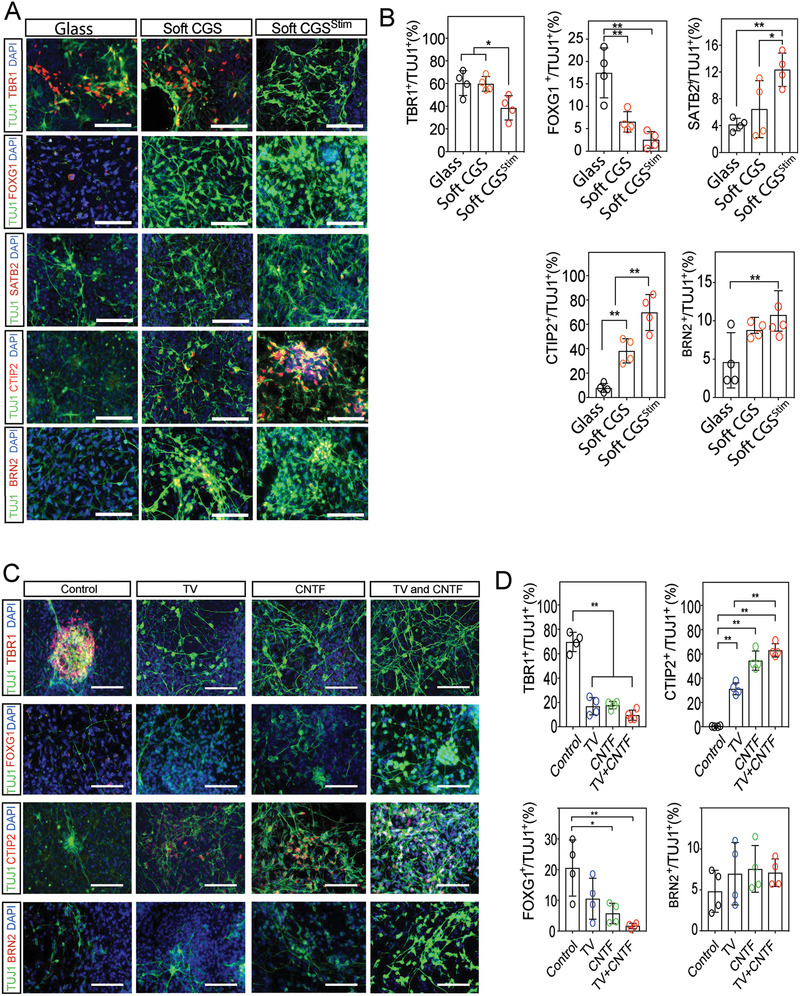
Characterization of Maturation of the iPSC derived Neurons. A) Representative images demonstrating co‐labeling of TUJ1 (green) and multiple post‐mitotic mature neuronal markers (red), TBR1, FOXG1, CTIP2, SATB2, and BRN2 on various substrates. B) Quantification of the percentage of TBR1^+^, FOXG1^+^, CTIP2^+^, SATB2^+^, and BRN2^+^ cells among TUJ1^+^ neurons. C) Representative images demonstrating co‐labeling of TUJ1 (green) and multiple post‐mitotic mature neuronal markers (red), TBR1, FOXG1, CTIP2, and BRN2 on glass substrate (control) in the presence of different treatments. D) Quantification of the percentage of TBR1^+^, FOXG1^+^, CTIP2^+^, and BRN2^+^ cells among TUJ1^+^ (green) neurons. A,C) Cell nuclei were counterstained with DAPI (blue). Scale bars indicate 100 µm. B,D) Analyzed using a one‐way ANOVA, followed by Tukey's HSD post hoc test with ^*^ *p* < 0.05 and ^**^ *p* < 0.01. Values represent the mean of independent experiments (*n* = 4); error bars, S.D.

Mature post‐mitotic markers were evaluated to further assess the maturity of the iPSC‐derived neurons. Because the dual SMAD differentiation protocol favors anterior dorsal fates, markers associated with the telencephalon (FOXG1) and cortical layers were evaluated. During corticogenesis, neurons of the adult cortex form cortical layers, such as TBR1^+^ (Layers VI, V, Layer 1 Cajal‐Retzius cells and subplate), CTIP2^+^ (Layers VI and V), SATB2^+^ (Layer II–IV), and BRN2 (Layers II–IV).^[^ ^1^, ^4^ ^]^ During development, the deeper cortical layers are generated first. We used these markers to identify more mature post‐mitotic differentiation from iPSCs in our system using markers that are expressed in rodent brain during different times of cortical development (**Figure** **7**). The enrichment of TBR1^+^ and FOXG1 neurons in the glass group suggest the generation of markers commonly associated with neurons that are generated earlier in development (Figure 7A). However, soft CGS culture and to a greater extent with electrical stimulation (soft CGS^Stim^) enabled generation of more mature neurons expressing post‐mitotic markers CTIP2^+^, SATB2, and BRN2 (Figure 7A,B). We observed that a much higher fraction of the cells become immature neurons (Tuj1) at the earlier time point. The fraction of cells that also acquire markers expressed only in more mature post‐mitotic neurons also increases (Satb2, Ctip, Brn2). Thus, our data demonstrate particularly efficient induction of more mature neurons within 14d of iPSC differentiation and introduce an approach to accelerate derivation of these neurons using mechanical and electrical cues.

To further characterize the iPSC‐derived neurons obtained by inhibiting RhoA and adding CNTF, the same post‐mitotic markers were analyzed. Addition of TV, CNTF, and TV+CNTF enabled the generation of neurons expressing CTIP2^+^ (Figure 7C,D). However, there was no generation of SATB2^+^ neurons or increase in BRN2^+^ neurons. Although neuronal differentiation by the addition of both factors may induce more mature post‐mitotic neurons, it appears mechanical and electrical stimulation likely affect multiple pathways to further accelerate the induction of neuron maturation.

## 3 Conclusion

This work developed a CGS platform modified with carbon nanofibers to apply mechanical and electrical stimulation to provide a method of efficient stem cell neuronal differentiation. A 3D, soft CGS (≈3 kPa) exposed to electrical stimulation rapidly generates iPSC‐derived neurons with a more mature, post‐mitotic identity and that have more mature electrophysiological properties by 14d of differentiation on the CGS. The described method has the additional advantage of being less reliant upon soluble factors (i.e., BDNF and NT3),^[^ ^1^, ^5^ ^]^ inhibitor cocktails (i.e., SMAD, Notch, or Wnt pathway inhibitors)^[^ ^4^, ^6^ ^]^ or viral vectors (i.e., NeuroD1, Ascl1 or Brn2)^[^ ^9^, ^40^, ^41^ ^]^ (Table S2, Supporting Information), which have inherent drawbacks (i.e., prolonged procedure duration, low yields, and viral contamination). Utilizing mechanical and electrical cues via our CGS platform appears to efficiently produce more mature neurons assessed both by markers expressed and through electrophysiological measurements of the iPSC‐differentiated neurons than many of the current protocols.

Notably, our results suggest two distinct pathways by which the combination of mechanical and electrical activity greatly affects human iPSC differentiation. The first mechanism involves stiffness‐dependent neuronal differentiation associated with the RhoA pathway. Varying the stiffness of the CGS affected cytoskeletal polymerization and regulated the RhoA signaling cascade. Mechanical feedback from intrinsic forces through cell–cell or cell–matrix junctions controls RhoA expression by simultaneously activating ROCK.^[^ ^42^ ^]^ The optimal stiffness used in these studies range from 2 to 3 kPa (soft CGS), similar to developing cortical tissue.^[^ ^43^ ^]^ Increased stiffness in CGS by lowering CNF content (1:10, ≈12 kPa) limited neuronal conversion of iPSCs. Its downstream pathways such as YAP and SMAD, which regulate the pluripotency of stem cells, were also controlled,^[^ ^18^, ^19^, ^20^ ^]^ further elucidating the importance of RhoA in stem cell differentiation and in a cell's response to mechanical stress. Future development of materials that have tunable stiffness is a valuable tool to optimize stem cell therapies and will help to further delineate how to modulate important differentiation pathways.

The second mechanism by which electrical stimulation enhanced neuronal conversion of iPSCs involves promoting trophic factor release (CNTF) and enables the transcriptional changes necessary for neuronal differentiation. To date, most of the literature on neuronal differentiation of iPSCs has concentrated on exogenously delivered neurotrophic factors (i.e., BDNF and NT3).^[^ ^1^, ^5^ ^]^ However, electrical activity plays an essential role in early development of the nervous system as the stimulation directly or indirectly regulates endogenous neurotrophic factor gene expressions including VEGFA, NT3, and BDNF.^[^ ^12^, ^25^ ^]^ Our work demonstrates that iPSCs respond to the electrical activity of their microenvironment, increasing endogenous CNTF release to enhance neuronal differentiation. If the CNTF pathway was inhibited, the rapid conversion of iPSCs was not observed. Our work adds to the prior literature which uses exogenously delivered CNTF to develop motor neurons and induce neuronal differentiation in retinal cells.^[^ ^6^, ^44^, ^45^ ^]^ The use of CNTF as described above shows to our knowledge a new efficient method to differentiate neuronal cortical cells using CNTF or electrical cues. The ability to identify important pathways from stem cell biology and act on those pathways is a powerful tool to develop new strategies for neuronal regeneration applications.

Cortical development occurs with deep layer neurons produced first, followed by upper layer neurons expressing a variety of cortical markers.^[^ ^1^, ^4^ ^]^ The capability to rapidly create iPSC‐derived, mature post‐mitotic neurons indicates effective neuronal maturation due to combined modalities of stimulation. Although animal in vivo models have been developed to investigate the regulatory role of neurons in various disease states including amyotrophic lateral sclerosis, spinal muscular atrophy, and addiction;^[^ ^46^, ^47^, ^48^ ^]^ such models do not necessarily represent human pathophysiology. By deriving iPSCs from patients with these disease states in our 3D, soft CGS, in vitro disease modeling and drug screening by more accelerated directed differentiation of iPSCs is possible. Further biological characterization is required to determine the exact characteristics of the post‐mitotic neurons that were formed, but our study demonstrated the 3D, soft CGS can be used to generate neurons with markers often observed in more mature cortical neurons.

The 3D CGS can be perturbed at various time points with electrical stimulation to observe the response to better understand neurologic disease states. This provides an advantage over traditional 2D, inert polymeric, and organoid systems to allow for continuous interactions with in vitro stem cells.^[^ ^10^ ^]^ These findings reinforce the concept that the combination of mechanical and electrical cues concurrently affects rapid neuronal conversion and emphasizes the need to often alter multiple pathways to enact change upon the nervous system. The 3D, conductive CGS allows for manipulation of the microenvironment of stem cells for regenerative and disease modeling strategies.

## 4 Experimental Section

##### Fabrication and Characterization of the CGS—Preparation of GOs

GOs were fabricated using the modified Improved Hummers method with modification.^[^ ^49^ ^]^ Briefly, 1 g of graphite flakes (Micro890, high purity graphite flake with a D50 particle size between 7–11 µm, 99%+ carbon purity) supplied from Ashbury Carbons (Asbury Carbon, NJ) was added into acidic solution (9:1, H_2_SO_4_:HNO_3_, Sigma‐Aldrich), and the solution was stirred without heat for 30 min. 6 g of potassium permanganate (KMnO_4_) (Sigma‐Aldrich, St. Louis, MO) was slowly added into the solution, and the solution was covered with tin foil, heated to 50 °C, and incubated overnight. 5 mL of H_2_O_2_ ice‐cold solution was added and stirred for 2 h. The solution was centrifuged at 4500 rpm for 45 min. The purification with HCl (0.1 m) and three consecutive washes with DI H_2_O were applied to remove unreacted carbon and metal ions. Collected GOs (2 mg mL^−1^) were stored at 4 °C.

##### Fabrication and Characterization of the CGS—Preparation of 3D‐nanoconfined conductive graphene scaffold (3D and 2D CGS)

CNFs (Sigma Aldrich) had a diameter ≈100 nm and length ≈20–200 µm (manufacturer's specifications). Varying ratios of CNFs (0, 1:10, 1:2, and 1:1) were suspended in GO solution and were sonicated using a bath sonicator (Branson Ultrasonic, Danbury, CT) for 1 h at 60 W followed by centrifugation at 800 rpm for 5 min (Allegra 25R, Beckman Coulter, Indianapolis, IN) to sediment CNF bundles. The concentrated GO:CNF suspensions were degassed to remove any bubbles. Reducing agents including sodium iodide and ascorbic acid, at a concentration of 10 wt% to induce self‐assembly of CGS, were added into suspensions; and it was poured into cylindrical molds. The suspensions formed the CGS at 80 °C within 24 h. The CGS had dimensions of 6 × 2 mm (diameter × height). To form a 2D CGS, the same steps were used to form 3D CGS. Subsequently, the CGS was placed between glass slides and then weight of 1 kg was applied for 1 h. The CGSs were neutralized by washing with deionized water until pH of supernatant equilibrated to 7. Collected CGSs were autoclaved and stored at 4 °C.

##### Fabrication and Characterization of the CGS—Characterization and measurement

Morphological properties were assessed by scanning electron microscopy (Zeiss Sigma FESEM). Specimens were sputter coated with Au–Pd, and attached to aluminum stabs with double‐sided copper tape. The morphological status was detected using In‐Lens secondary electron detector with accelerating voltage 10 kV. Rheological properties of the CGS were measured using a stress‐controlled mechanical analyzer (AR‐G2, TA instrument, New Castle, DE). Static compression tests were performed at 37 °C in the strain ramp mode with a ramp rate of 5 µm s^−1^. The dimensions of the tested samples were 6 × 2 mm (*D* × *T*, *D*: diameter; *t*: thickness). The compressive stress of the CGS was derived from the force divided by the cross‐sectional area of the scaffolds. The bulk electrical conductivities of cylindrical CGSs were measured by the four‐probe method with metal electrodes attached to the ends of samples. The electric field behavior in the CGS was simulated by ANSYS Maxwell static 3D electromagnetic finite element simulation of the electric field distribution (ANSYS HFSS, ANSYS Corp., Canonsburg, PA). In particular, an AC conduction analysis was performed to plot the electric field distribution in the CGS. The simulator conducts the Maxwell equations in a defined region of space with diameter (OD: 6 mm), height (*H*: 2 mm), and square electrodes (1 × 1 mm). The simulator calculated electric field distribution (mV mm^−1^) while the current density ranging from 0 to ±2 V gradually increased.

##### Neuronal Differentiation of iPSCs on CGS

All stem cell procedures were approved by Stanford's Stem Cell Research Oversight committee (SCRO: 616). Culture of the human iPSC line (human iPSC was generated from BJ fibroblasts using mRNA reprogramming factor sets leading to the overexpression of OCT4, SOX2, KLF4, and c‐MYC as described previously) was carried out on a matrigel‐treated 6‐well plate in mTeSR.^[^ ^50^ ^]^ Cells were incubated at 37 °C in 5% CO_2_, and passaged every 5–7d with Accutase (Innovative Cell Technologies, San Diego, CA). iPSCs from passage 51–55 were used in these studies.

##### Neuronal Differentiation of iPSCs on CGS—Differentiation of iPSCs to neural precursor cells

Human iPSC‐derived neural precursor cells were generated using defined conditions with modification to previously reported protocols.^[^ ^1^, ^5^ ^]^

NPC differentiation base medium formulation: DMEM/F12 (50%), Neurobasal (50%), N2‐MAX (1%), B27 (1%) nonessential amino acids (NEAA) (1%), GlutaMAX (1%), 2‐mercaptoethanol (0.1 × 10^−3^ m), penicillin/streptomycin (P/S, 1% v/v) supplemented with SMAD inhibitors such as Dorsomorphin (1 × 10^−6^ m) and SB431542 (1 × 10^−6^ m).

NPC maintenance base medium formulation: DMEM/F12 (50%), Neurobasal (50%), N2‐MAX (1%), B27 (1%) nonessential amino acids (NEAA) (1%), GlutaMAX (1%), 2‐mercaptoethanol (0.1 × 10^−3^ m), penicillin/streptomycin (P/S, 1% v/v) supplemented with bFGF (20 ng mL^−1^) and EGF (20 ng mL^−1^).

Day (0): Human iPSCs at ≈90% confluency were first washed with room temperature 1× DPBS without Ca^2+^ and Mg^2+^ once. Wash was aspirated and cells were primed by the treatment with NPC differentiation base medium for 7d (4 mL per 6‐well plate) under standard cell culture condition (37 °C, 5% CO_2_). Fresh medium was replenished every 24 h.

Day (7): After the induction procedure, NPCs were washed with DPBS once. The cells were then detached from the plates with Accutase (1 mL per well) and placed in incubator (37 °C). After 5 min, the side and bottom of the plate was gently rubbed to dislodge the cells from the plate surface. Then cells were collected into a 15 mL conical tube using a 10 mL serological pipette and 9 mL of DMEM/F12 containing RhoA/ROCK inhibitor, TV (2 × 10^−6^ m) was added. Cells were centrifuged at 1200 rpm for 5 min at room temperature. After centrifugation, supernatant was aspirated and the cell pellet was resuspended in NPC maintenance medium + TV (2 × 10^−6^ m). Cells were re‐plated on CGS (ranging from 3 to 12 kPa) (100 000 cells cm^−2^) previously coated with 10 µg mL^−1^ poly‐l‐ornithine solution (Sigma‐Aldrich, St. Louis, MO) and 4 µg mL^−1^ laminin (Sigma‐Aldrich, St. Louis, MO) in PBS. Then scaffolds with the cells were incubated under standard cell culture conditions (37 °C, 5% CO_2_) for 24 h.

##### Differentiation of NPCs to Neurons Using Combination of Mechanical and Electrical Stimulation

Neuronal differentiation base medium formulation:DMEM/F12 (50%), Neurobasal (50%), N2‐MAX (1%), B27 (1%) nonessential amino acids (NEAA) (1%), GlutaMAX (1%), 2‐mercaptoethanol (0.1 × 10^−3^ m), penicillin/streptomycin (P/S, 1% v/v).

Day (1; or day 8 from iPSCs)—Electrical Stimulation: The media was aspirated and the scaffold was transferred to electrical stimulation chamber. In vitro iPSC‐electrical stimulation was applied by means of an alternating current‐electrical stimulation, custom built cell culture chamber (Figure 1a). The chamber consists of parallel indium tin oxide (ITO) patterned electrodes, separated by a distance of 3 mm. The electrodes were connected to a waveform generator (Keysight, Englewood, CO). The cells cultured on CGS were exposed to electrical stimulation for 1 h. After the stimulation was applied, the scaffolds were carefully transferred to a 24‐well plate with 1 mL of fresh medium.

Day (2; or day 9 from iPSCs)—Cell viability after electrical stimulation: To optimize the cell viability after the stimulation, the metabolic rate of reasazurin (Life Technologies, Carlsbad, CA) and Live/Dead staining (Life Technologies, Carlsbad, CA) were performed. For the metabolic assay, the media containing 0.5% of reasazurin was added to the cell culture. After a 6 h incubation, the media (10 µL) was collected and mixed with 990 µL of DPBS. The fluorescence intensity of solution was recorded using a multiplate reader (Ex: 535 nm; Em: 585 nm). Lysed cells were utilized as a positive control. The same density of cells cultured on a TC plate coated with the same coating substances including PLO/Laminin were used as a negative control. For the Live/Dead assay, the supernatant was aspirated and the cells were incubated with Live/Dead solution. After staining, the cells were visualized by fluorescence microscopy (Keyence All‐in‐One Fluorescence Microscope (BZ‐X700) (Keyence corp., Itasca, IL) (Live, Ex: 485 nm; Em: 535 nm and Dead, Ex: 535 nm; Em: 565 nm).

##### Immunocytofluorescence

On day 7 (or day 14 from iPSCs), cells were fixed with 4% paraformaldehyde (electron microscopy sciences) for 1 min and then permeabilized and blocked with blocking buffer (0.1% Triton X‐100, 1% BSA (Fisher BioReagents, Santa Clara, CA), 5% normal goat serum (NGS, Invitrogen, Waltham, MA) for 1 h at room temperature. Primary antibodies (listed in Key Resource, Table S3, Supporting Information) were incubated in blocking buffer at 4 °C overnight, followed by three 15 min PBS washes and detected by secondary antibodies (Alexa Flour 488, 555, or 647, Life Technologies). Samples were counter‐stained with DAPI (Sigma‐Aldrich, St. Louis, MO) to visualize nuclei and mounted with Fluoromount Aqueous Medium (Sigma‐Aldrich, St. Louis, MO) before imaging. Samples were imaged on a Keyence All‐in‐One Fluorescence Microscope (BZ‐X700) (Keyence corp., Itasca, IL) using 20× or 60× objectives. Image of cells on CGSs are presented as maximum intensity projections of z‐stacks generated from BZ‐X Analyzer. The neuronal differentiation efficacy of iPSCs was quantified by counting the total number of TUJ1‐positive cells with neuronal morphology. The number of TUJ1‐positive cells was divided by the total number of cell nuclei (DAPI‐positive) to demonstrate the percentage of neuronal differentiation. Calculations were performed using randomly selected four different locations from four individual samples at 20× magnification. In addition, co‐localization of proteins such as YAP and p‐SMAD in cell nucleus was quantified by counting the number of co‐positive for DAPI. The overall of pattern of those staining was categorized as nuclear only or cytoplasmic only through use of z‐stacks. Z‐stacked microscopy images of two different channels for YAP/p‐SMAD and DAPI were merged into one image to determine overlap. All image quantification for percentages of marker‐positive cells was performed by a blinded individual via ImageJ and manual counting.

##### RNA Isolation and Quantitative Real‐Time PCR (qRT‐PCR) Analysis

Total RNA was extracted from cells using a Qiagen RNeasy Plus Micro Kit (Qiagen, Germantown, MD). After accomplishing first‐strand cDNA synthesis by iScript cDNA Synthesis Kit (Bio‐Rad, Hercules, CA), quantitative real‐time polymerase chain reaction (qRT‐PCR) was performed with Taqman‐polymerase and primers (Qiagen, Germantown, MD) for gene expression analysis. qRT‐PCR was carried out on a QuantStduio 6 Flex Real‐Time PCR System (ThermoFisher, Waltham, MA). The Delta–Delta CT method was utilized for relative expression levels with GAPDH as a housekeeping gene and iPSCs grown on glass as references. Taqman primers used in these studies are listed in Key Resource, Table S3 (Supporting Information).

##### ELISA Analysis

For CNTF ELISA, the conditioned media was collected at 24 h after exposure to electrical stimulation. Samples were assayed by CNTF Development kit from Peprotech (Peprotech, Rocky Hill, NJ) according to the manufacturer's instructions.

##### Factor Addition/KD Study

Day 1 (or day 8 from iPSCs):To perform the factor addition study, human NPCs cultured on a precoated glass substrate (PLO/Laminin with 100 000 cells cm^−2^) were conditioned with RhoA/ROCK inhibitor (Thiazovivin, 2 × 10^−6^ m), CNTF (1 ng mL^−1^), or a combination of both. Cell cultures were maintained for 7d with a medium change every other day. After the culture, cells were utilized for subsequent analysis including immunocytofluorescence and qRT‐PCR analysis.

Generation of CNTF^KD^ iPSCs:To conduct the KD study, CNTF expression in human iPSCs was inhibited using CNTF shRNA (SC‐41921‐V) and control scrambled shRNA lentiviral particles (SC‐108080) purchased from Santa Cruz Biotechnology (Santa Cruz Biotechnology, Santa Cruz, CA). Briefly, iPSCs were cultured in mTeSR on matrigel‐coated 6‐well plates until 30% confluent then treated with 20 µL concentrated viral particles containing shCNTF or the nontargeting control (scrambled, SC) overnight (day 0). The viral particle‐containing medium was removed and cells were allowed to recover (day 1) before selecting with 0.5 µg mL^−1^ puromycin (day 2). Cells were cultured continuously in mTeSR with puromycin for 7d total. Cell samples were either harvested for qRT‐PCR, immunocytofluorescence, and in‐cell western blot analysis to monitor CNTF knockdown efficiency. After the selection was completed, CNTF knockdown cells were utilized for subsequent study and analysis as described above. For the in‐cell western assay, the subcellular level of CNTF was quantified in situ using infrared (IR) intensity. After the inhibition of CNTF, the cells were plated in a 96‐well plate (20 000 cells per well) and were immunolabeled with an IR‐conjugated secondary antibody using the standard immunocytofluorescence protocol. After the completion of the staining procedure, the plate was imaged using an Odyssey Fc IR imaging system (LiCor, Lincoln, NE). CNTF intensity was normalized to GAPDH expression by using the Odysset CLx Image Studio Analysis software. After thorough analysis, CNTF^KD^ iPSCs were primed to NPC differentiation medium for 7d and then the cells were further utilized for the study of mechanical and electrical impact on neuronal differentiation on the CGS by following the protocol as described above.

##### Electrophysiology

On day 7 and 14 (or 14 and 21d from iPSCs), electrophysiological properties of the differentiated neurons on the CGS were investigated. The cultured iPSC‐derived neurons were transferred from a 37 °C incubator to the recording chamber, superfused with artificial cerebrospinal fluid (ACSF) containing 125 × 10^−3^ m NaCl, 2.5 × 10^−3^ m KCl, 2 × 10^−3^ m CaCl_2_, 1.25 × 10^−3^ m NaH_2_PO_4_, 1 × 10^−3^ m MgCl_2_, 25 × 10^−3^ m NaHCO_3_, and 15 × 10^−3^ m d‐glucose (300–305 mOsm) at a rate of 2–4 mL min^−1^. All solutions were saturated with 95% O_2_ and 5% CO_2_. The iPSC‐derived neurons were recorded at room temperature (20–22 °C) within 1 h after transferring. Due to the opaque property of the CGS substrate, which prevented using regular differential interference contrast (DIC) optics, epi‐fluorescence signal‐guided whole‐cell patch‐clamp recording was performed. eGPF‐expressing iPSCs were identified with a water‐immersion objective lens (60×, NA = 1.1; Olympus, Japan) mounted on an upright microscope (Olympus BX‐51) equipped with a mercury light source, and appropriate filter sets (470–490 nm for excitation; 515–550 nm for emission). To visualize the whole‐cell patch‐clamp pipette (3–5 MΩ), 2–5 × 10^−6^ m of Alexa Fluor 488 (Invitrogen, USA) was included in the internal solution containing 135 × 10^−3^ m KCH_3_SO_3_, 5 × 10^−3^ m KCl, 10 × 10^−3^ m HEPES, 8 × 10^−3^ m Na_2_‐phosphocreatine, 0.3 × 10^−3^ m Na_2_GTP, 4 × 10^−3^ m MgATP, 0.1 × 10^−3^ m CaCl_2_, 1 × 10^−3^ m EGTA (pH 7.2–7.3, 285–290 mOsm, pH was adjusted with KOH).

For voltage clamp recording, the series and input resistance of the iPSC‐derived neurons was measured by injection of hyperpolarizing pulses (−5 mV, 100 ms). The initial series resistances were <20 MΩ. In current clamp recording mode, a bridge balance was applied to compensate series resistance. Resting membrane potential was adjusted to −70 mV via somatic current injection. Current steps (800 ms, 5–60 pA with 5 pA step size) were injected to the iPSC‐derived neurons to test the membrane properties and to evoke action potentials. Recordings were obtained with a Multiclamp 700B (Molecular Devices, USA). Signals were filtered at 2.2 kHz and digitized at 10 kHz with NI PCIe‐6259 card (National Instruments). In some patch‐clamp recordings, two‐photon imaging was performed to reveal the morphology of the Alexa Fluor 488 (2 ×10^−6^–5 ×10^−6^ m) filled iPSCs (Figure 7b,c) with a custom built two‐photon laser‐scanning microscope equipped with a mode‐locked tunable (690–1040 nm) Ti:sapphire laser Mai Tai eHP (Spectra‐Physics, USA) tuned to 925 nm. The electrophysiology and imaging data were acquired with custom‐made software written in Matlab (Mathworks) described previously.^[^ ^51^ ^]^ The individual making the measurements was blinded from the groups.

##### Quantification and Statistical Analysis

All the data are presented as the mean ± standard deviation (SD) of four independent experiments. The *n* values indicate the number of independent experiments conducted or the number of individual experiments. An analysis of variance (ANOVA) test was used for multicomponent comparisons (*n* > 3 independent variables) after the normal distribution was confirmed using Shapiro‐Wilk normality test. Tukey post hoc analysis was performed to investigate the differences between variables. Image analysis, cell counting, and electrophysiological analysis were blinded and performed by independent investigators. The statistical parameters are summarized in Table S4 in the Supporting Information.

## Conflict of Interest

The authors declare no conflict of interest.

## Author Contributions

B.O. and P.M.G. conceived the concept, designed the experiments, analyzed and interpreted the data, and wrote the manuscript. Y.‐W.W. and J.D. designed and performed electrophysiological experiments related to neuronal culture on the CGS. J.D. provided critical insights into manuscript writing. V.S. and V.L. assisted with stem cell culture and fabrication of CGS and its characterization using FT‐IR and XPS.

## Supporting information

Supporting Information

## Data Availability

The data that support the plots within this paper and other findings of this study are available from the corresponding author on reasonable request.
