# Supplementary material for: Modulating the Electrical and Mechanical Microenvironment to Guide Neuronal Stem Cell Differentiation
Source: Adv Sci (Weinh). 2021 Feb 18;8(7):2002112. doi: 10.1002/advs.202002112 (PMC8025039; doi:10.1002/advs.202002112)
Supplement: Supplementary file 1 — Supporting Information [file ADVS-8-2002112-s001.pdf]

© 2020 Wiley-VCH GmbH

ADVANCED SCIENCE
Open Access

Supporting Information

for *Adv. Sci.*, DOI: 10.1002/advs.202002112

Modulating the Electrical and Mechanical Microenvironment to Guide Neuronal Stem Cell Differentiation

Dr. Byeongtaek Oh[1], Dr. Yu-Wei Wu[2,3], Vishal Swaminathan[1], Vivek Lam[1], Prof. Jun Ding[1,2], Prof. Paul M. George[1*]*

SUPPLEMENTARY INFORMATION

Type of Scaffold	E (kPa)	ρ (S/m)	Proportion of Tuj1+ Cells (%)	
			(-) Stimulation	(+) Stimulation
Glass			9.3±4.5	N/A
CGS (without CNF)	9.64±0.25	0.2±0.01	8.1±1.3	12.5±2.6
CGS (1:10)	8.69±0.56	0.21±0.01	12.6±3.4	19.7±8.5
CGS (1:2)	6.10±0.7	0.22±0.03	18.8±3.9	41.8±15.6
CGS (1:1)	3.11±0.6	0.21±0.02	39.2±6.8	75.9±3.4

Table S1. Characterization of CGS substrates.

Proportion of Tuj1-positive cells in culture on varying substrates with electrical stimulation (±800 mV, 100 Hz for 1 hr). Data are presented as Mean±S.D. (n=4). *E* indicates matrix stiffness; ρ indicates electrical conductivity.

Category	Targeted Cells	INH	GFs	*E* (kPa)	ST	Neuron (%)	Days	REF
Inhibitors	Cortical Neurons	DOR and SB	None	TC	NA	≥70%	70 d	[1]
Mechanical cues Trophic Factors	Motor Neurons	L+SB (Initial 7d)	BDNF IGF	≤5 kPa	NA	17 %	23 d	[18]
Mechanical cues	Neurons	NA	None	≤0.3 kPa	NA	≥80 %	19 d	[14]
3D Organoid Culture	Neurons	DOR+SB (Initial 7d)	BDNF, NT3	NA	NA	≥80 %	49 d	[5]
Trophic Factor Inhibitor	Neurons	DOR and SB	BDNF, GDNF	TC	NA	≥70%	70 d	[13]
Trophic Factor Inhibitor	Cortical Neurons	L+SB+P/S/D	BDNF	TC	NA	≥70%	13 d	[4]
Trophic Factor Inhibitor	Motor Neurons	L+SB+P/S/D	BDNF GDNF	TC	NA	≥70%	14 d	[6]
Viral infection	Excitatory Neurons	NA	BDNF NT3	TC	NA	≥90%	14 d	[7]
Mechanical and Electrical cues	Cortical Neurons	DOR and SB (Initial 7d)	None	≤3 kPa	Electrical stimulation	≥80 %	14 d	CS

Table S2. Summary and comparison of neural differentiation efficacy to previously published studies.

INH: Inhibitors (DOR: Dorsomorphin, SB: SB431542, L: LDN193189, P/S/D: PD0325901, SU5402, DAPT cocktail), GFs: growth factors (BDNF: brain-derived neurotrophic factor, IGF: insulin-like growth factor, GDNF: glial cell -derived neurotrophic factor, and NT3: Neurotrophin-3), E: Matrix stiffness, ST: Stimulation and REF: Reference. NA indicates not applied. CS: Current study.

REAGENT or RESOURCE	SOURCE	IDENTIFIER
**Antibodies**		
Mouse anti-TUJ1	Neuromics	MO15013
Chicken anti-MAP2	Invitrogen	PA-11671
Rabbit anti-TBR1	Abcam	AB31940
Rabbit anti-CTIP2	Abcam	AB28448
Mouse anti-SATB2	Abcam	AB51502
Rabbit anti-GFAP	EMD Millipore	AB5804
Rabbit anti-RhoA	Invitrogen	OSR00266W
Mouse anti-YAP	Santa Cruz Biotech	SC 101199
Rabbit anti-p-SMAD	EMD Millipore	AB3848-I
Rabbit anti-Ki67	Invitrogen	PA-121520
Mouse anti-Nestin	Invitrogen	MA-1110
Rabbit anti-CNTF	Novus Biologicals	NBP183277
Rabbit anti-Oct4	Invitrogen	701756
Mouse anti-SSEA4	Invitrogen	MA1021
Mouse anti-GAPDH	Novus Biologicals	NBP2-37828
Alexa Flour 488 Goat anti-rabbit	Thermo Scientific	A11034
Alexa Flour 488 Goat anti-mouse	Thermo Scientific	A32732
Alexa Flour 568 Goat anti-rabbit	Thermo Scientific	A11036
Alexa Flour 568 Goat anti-chicken	Thermo Scientific	A11041
Alexa Flour 568 Goat anti-mouse	Thermo Scientific	A11004
Alexa Flour 647 Goat anti-chicken	Thermo Scientific	A21449
Alexa Flour 647 Goat anti-rabbit	Thermo Scientific	A32733
IRDye® 800CW Goat anti-Mouse	LI-COR	P/N 925-32210
**IRDye® 680LT Goat anti-Rabbit**	LI-COR	P/N 925-32210
**Biological Samples**		
Bovine serum albumin (BSA)	Fisher BioReagents	BP9706100
Normal goat serum (NGS)	Invitrogen	31873
CNTF shRNA Lentiviral particles	Santa Cruz Biotech	SC-41921-V
Control shRNA Lentiviral particles	Santa Cruz Biotech	SC-108080
GFP Control Lentiviral particles	Santa Cruz Biotech	SC-108084
**Chemicals, inhibitors, and Recombinant Protein**		
Graphite	Ashbury carbon	Micro890
Carbon nanofiber	Sigma Aldrich	719781
Sulfuric acid	Fisher Chemical	A510-P212
Phosphoric acid	Fisher Chemical	A242-1
Ascorbic acid	Fisher Chemical	A61-100
Sodium iodide	Fisher Chemical	S324-100
Hydrogen peroxide solution (30 wt%)	Acros Organics	AC411885000
Thiazovivin	Selleck Chemical	50-753-2
Recombinant Human CNTF	Peprotech	450-13
DMEM/F-12 Medium	Gibco	11330057
Neurobasal Medium	Gibco	21103049
B27 Supplement	Gibco	17504044
N2 MAX Supplement	R&D system	AR009
Non-essential amino acid	GE Healthcare	SH30238.01
GlutaMAX	Gibco	35050061
GFR membrane matrix	Corning	354230
Poly-L-ornithine hydrobromide	Sigma Aldrich	P3655
Laminin	Sigma Aldrich	L2020
Penicillin-Streptomycin	Gibco	15140122
Dorsomorhoin	Apexbio	50-101-3624

SB431542	Apexbio	50-101-2514
**Taqman Gene Expression Probes**		
GAPDH		Hs02758991_g1
Tubb3		Hs00801390_s1
Map2		Hs00258900_m1
GFAP		Hs00909233_m1
Nes		Hs04187831_g1
Rhoa		Hs00357608_m1
Syn1		Hs00199577_m1
Cntf		Hs04194755_s1
Nt3		Hs00267375_s1
Bdnf		Hs02718934_s1
Vegfa		Hs00900055_m1
nNos		Hs00167223_m1
Mmp9		Hs00957562_m1
Mmp14		Hs01037003_g1
**Software**		
GraphPad Prism	GraphPad	
ImageJ	NIH	
ANSYS HFSS	ANSYS Corp.	

Table S3. Key resource table.

Figure	Test	*F*-Value	Condition	*P*-value
Figure 1c	ANOVA followed by Tukey	28.45	Control (0) vs. CNF:GO (1:1)	<0.0001
Figure 1e	ANOVA followed by Tukey	7.374	Glass vs. Stiff CGS	0.8174
Figure 1e	ANOVA followed by Tukey	7.374	Glass vs. Soft CGS	0.0374
Figure 1e	ANOVA followed by Tukey	7.374	Stiff CGS vs. Soft CGS	0.0146
Figure 1f	ANOVA followed by Tukey	202.4	Glass vs. Stiff CGS	0.0736
Figure 1f	ANOVA followed by Tukey	202.4	Glass vs. Soft CGS	<0.0001
Figure 1f	ANOVA followed by Tukey	202.4	Stiff CGS vs. Soft CGS	<0.0001
Figure 1h	ANOVA followed by Tukey	33.36	Glass vs. Stiff CGS	0.4748
Figure 1h	ANOVA followed by Tukey	33.36	Glass vs. Soft CGS	<0.0001
Figure 1h	ANOVA followed by Tukey	33.36	Stiff CGS vs. Soft CGS	0.0003
Figure 1i	ANOVA followed by Tukey	21.76	Glass vs. Stiff CGS	0.9644
Figure 1i	ANOVA followed by Tukey	21.76	Glass vs. Soft CGS	0.0006
Figure 1i	ANOVA followed by Tukey	21.76	Stiff CGS vs. Soft CGS	0.0009
Figure 2c (TUJ1)	ANOVA followed by Tukey	33.32	Glass vs. Soft CGS	0.0054
Figure 2c (TUJ1)	ANOVA followed by Tukey	33.32	Glass vs. Soft CGS^{Stim}	<0.0001
Figure 2c (TUJ1)	ANOVA followed by Tukey	33.32	Soft CGS vs. Soft CGS^{Stim}	0.0091
Figure 2c (MAP2)	ANOVA followed by Tukey	36.65	Glass vs. Soft CGS	0.047
Figure 2c (MAP2)	ANOVA followed by Tukey	36.65	Glass vs. Soft CGS^{Stim}	<0.0001
Figure 2c (MAP2)	ANOVA followed by Tukey	36.65	Soft CGS vs. Soft CGS^{Stim}	0.0008
Figure 2e (TBR1)	ANOVA followed by Tukey	6.721	Glass vs. Soft CGS	0.9956
Figure 2e (TBR1)	ANOVA followed by Tukey	6.721	Glass vs. Soft CGS^{Stim}	0.0257
Figure 2e (TBR1)	ANOVA followed by Tukey	6.721	Soft CGS vs. Soft CGS^{Stim}	0.0296
Figure 2e (CTIP2)	ANOVA followed by Tukey	34.85	Glass vs. Soft CGS	0.0067
Figure 2e (CTIP2)	ANOVA followed by Tukey	34.85	Glass vs. Soft CGS^{Stim}	<0.0001
Figure 2e (CTIP2)	ANOVA followed by Tukey	34.85	Soft CGS vs. Soft CGS^{Stim}	0.0055
Figure 2e (SATB2)	ANOVA followed by Tukey	8.452	Glass vs. Soft CGS	0.5269
Figure 2e (SATB2)	ANOVA followed by Tukey	8.452	Glass vs. Soft CGS^{Stim}	0.008
Figure 2e (SATB2)	ANOVA followed by Tukey	8.452	Soft CGS vs. Soft CGS^{Stim}	0.0445
Figure 2k	Unpaired t-test	N/A	Soft CGS vs. Soft CGS^{Stim}	0.0041
Figure 2l	Unpaired t-test	N/A	Soft CGS vs. Soft CGS^{Stim}	0.0267
Figure 3b (*Nestin*)	ANOVA followed by Tukey	33.32	Glass vs. Soft CGS	0.0054
Figure 3b (*Nestin*)	ANOVA followed by Tukey	23.7	Glass vs. Soft CGS^{Stim}	<0.0001
Figure 3b (*Nestin*)	ANOVA followed by Tukey	33.32	Soft CGS vs. Soft CGS^{Stim}	0.0091
Figure 3b (*Tubb3*)	ANOVA followed by Tukey	23.7	Glass vs. Soft CGS	0.5282
Figure 3b (*Tubb3*)	ANOVA followed by Tukey	23.7	Glass vs. Soft CGS^{Stim}	0.0003
Figure 3b (*Tubb3*)	ANOVA followed by Tukey	23.7	Soft CGS vs. Soft CGS^{Stim}	0.0012
Figure 3b (*Map2*)	ANOVA followed by Tukey	33.83	Glass vs. Soft CGS	0.4020
Figure 3b (*Map2*)	ANOVA followed by Tukey	33.83	Glass vs. Soft CGS^{Stim}	<0.0001
Figure 3b (*Map2*)	ANOVA followed by Tukey	33.83	Soft CGS vs. Soft CGS^{Stim}	0.0004
Figure 3b (*Syn1*)	ANOVA followed by Tukey	69.67	Glass vs. Soft CGS	0.0385
Figure 3b (*Syn1*)	ANOVA followed by Tukey	69.67	Glass vs. Soft CGS^{Stim}	<0.0001
Figure 3b (*Syn1*)	ANOVA followed by Tukey	69.67	Soft CGS vs. Soft CGS^{Stim}	<0.0001
Figure 3c (*Cntf*)	ANOVA followed by Tukey	19.95	Glass vs. Soft CGS	0.9730
Figure 3c (*Cntf*)	ANOVA followed by Tukey	19.95	Glass vs. Soft CGS^{Stim}	0.0009
Figure 3c (*Cntf*)	ANOVA followed by Tukey	19.95	Soft CGS vs. Soft CGS^{Stim}	0.0012
Figure 3d	ANOVA followed by Tukey	13.27	Glass vs. Soft CGS	0.4474
Figure 3d	ANOVA followed by Tukey	13.27	Glass vs. Soft CGS^{Stim}	0.0020
Figure 3d	ANOVA followed by Tukey	13.27	Soft CGS vs. Soft CGS^{Stim}	0.0124
Figure 4c (*Nestin*)	ANOVA followed by Tukey	10.75	Control vs. TV+CNTF	0.0017
Figure 4c (*Tubb3*)	ANOVA followed by Tukey	3.95	Control vs. CNTF	0.0354
Figure 4c (*Tubb3*)	ANOVA followed by Tukey	3.95	Control vs. TV+CNTF	0.0434
Figure 4c (*Map2*)	ANOVA followed by Tukey	17.17	Control vs. TV+CNTF	0.0001
Figure 4c (*Map2*)	ANOVA followed by Tukey	17.17	TV vs. TV+CNTF	0.0009
Figure 4c (*Map2*)	ANOVA followed by Tukey	17.17	CNTF vs. TV+CNTF	0.0007
Figure 4c (*Syn1*)	ANOVA followed by Tukey	29.36	Control vs. TV+CNTF	<0.0001
Figure 4c (*Syn1*)	ANOVA followed by Tukey	29.36	TV vs. TV+CNTF	0.0004
Figure 4c (*Syn1*)	ANOVA followed by Tukey	29.36	CNTF vs. TV+CNTF	0.0020
Figure 4d (TUJ1)	ANOVA followed by Tukey	23.1	Control vs. TV	0.0009
Figure 4d (TUJ1)	ANOVA followed by Tukey	23.1	Control vs. CNTF	0.0003
Figure 4d (TUJ1)	ANOVA followed by Tukey	23.1	Control vs. TV+CNTF	<0.0001
Figure 4d (MAP2)	ANOVA followed by Tukey	30.71	Control vs. TV	0.0113

Figure 4d (MAP2)	ANOVA followed by Tukey	30.71	Control vs. CNTF	0.0002
Figure 4d (MAP2)	ANOVA followed by Tukey	30.71	Control vs. TV+CNTF	<0.0001
Figure 4d (MAP2)	ANOVA followed by Tukey	30.71	TV vs. TV+CNTF	0.0008
Figure 4f (TBR1)	ANOVA followed by Tukey	87.71	Control vs. TV	<0.0001
Figure 4f (TBR1)	ANOVA followed by Tukey	87.71	Control vs. CNTF	<0.0001
Figure 4f (TBR1)	ANOVA followed by Tukey	87.71	Control vs. TV+CNTF	<0.0001
Figure 4f (CTIP2)	ANOVA followed by Tukey	105.7	Control vs. TV	<0.0001
Figure 4f (CTIP2)	ANOVA followed by Tukey	105.7	Control vs. CNTF	<0.0001
Figure 4f (CTIP2)	ANOVA followed by Tukey	105.7	Control vs. TV+CNTF	0.0001
Figure 4f (CTIP2)	ANOVA followed by Tukey	105.7	TV vs. TV+CNTF	<0.0001
Figure 4h	ANOVA followed by Tukey	2.382	Control vs. TV+CNTF	0.0419
Figure 5b	ANOVA followed by Tukey	104.2	Negative vs. Scramble$^{KD+Stim}$	<0.0001
Figure 5b	ANOVA followed by Tukey	104.2	Scramble^{KD} vs. Scramble$^{KD+Stim}$	<0.0001
Figure 5b	ANOVA followed by Tukey	104.2	CNTF^{KD} vs. Scramble$^{KD+Stim}$	<0.0001
Figure 5b	ANOVA followed by Tukey	104.2	CNTF$^{KD+Stim}$ vs. Scramble$^{KD+Stim}$	<0.0001
Figure 5b	ANOVA followed by Tukey	104.2	CNTF^{KD} vs. CNTF$^{KD+Stim}$	>0.9999
Figure 5c	ANOVA followed by Tukey	112	Negative vs. Scramble$^{KD+Stim}$	<0.0001
Figure 5c	ANOVA followed by Tukey	112	Scramble^{KD} vs. Scramble$^{KD+Stim}$	<0.0001
Figure 5c	ANOVA followed by Tukey	112	CNTF^{KD} vs. Scramble$^{KD+Stim}$	<0.0001
Figure 5c	ANOVA followed by Tukey	112	CNTF$^{KD+Stim}$ vs. Scramble$^{KD+Stim}$	<0.0001
Figure 5c	ANOVA followed by Tukey	112	CNTF^{KD} vs. CNTF$^{KD+Stim}$	0.993
Figure 5e	ANOVA followed by Tukey	19	Scramble^{KD} vs. Scramble$^{KD+Stim}$	0.0008
Figure 5e	ANOVA followed by Tukey	19	CNTF^{KD} vs. Scramble$^{KD+Stim}$	<0.0001
Figure 5e	ANOVA followed by Tukey	19	CNTF$^{KD+Stim}$ vs. Scramble$^{KD+Stim}$	0.0004
Figure 5e	ANOVA followed by Tukey	19	CNTF^{KD} vs. CNTF$^{KD+Stim}$	0.7714
Figure 5g	Unpaired t-test	N/A	Scramble$^{KD+Stim}$ vs. CNTF$^{KD+Stim}$	0.0323
Figure S1a	ANOVA followed by Tukey	228.4	Glass vs. PS substrate	>0.9999
Figure S1a	ANOVA followed by Tukey	228.4	Glass vs. Stiff CGS	<0.0001
Figure S1a	ANOVA followed by Tukey	228.4	Glass vs. Soft CGS	<0.0001
Figure S1a	ANOVA followed by Tukey	228.4	PS substrate vs. Stiff CGS	<0.0001
Figure S1a	ANOVA followed by Tukey	228.4	PS substrate vs. Soft CGS	<0.0001
Figure S1k	ANOVA followed by Tukey	23.15	Glass vs. Soft CGS	0.0003
Figure S1k	ANOVA followed by Tukey	23.15	Stiff CGS vs. Soft CGS	0.0015
Figure S2f (TUJ1)	ANOVA followed by Tukey	15.66	Glass vs. Stiff CGS^{Stim}	0.0017
Figure S2f (TUJ1)	ANOVA followed by Tukey	15.66	Stiff CGS vs. Stiff CGS^{Stim}	0.0036
Figure S2f (MAP2)	ANOVA followed by Tukey	43.93	Glass vs. Stiff CGS^{Stim}	<0.0001
Figure S2f (MAP2)	ANOVA followed by Tukey	43.93	Stiff CGS vs. Stiff CGS^{Stim}	<0.0001
Figure S3a (*Nestin*)	ANOVA followed by Tukey	10.19	Glass vs. Stiff CGS^{Stim}	0.0345
Figure S3a (*Nestin*)	ANOVA followed by Tukey	10.19	Stiff CGS vs. Stiff CGS^{Stim}	0.0043
Figure S3a (*Tubb3*)	ANOVA followed by Tukey	8.631	Glass vs. Stiff CGS^{Stim}	0.05
Figure S3a (*Tubb3*)	ANOVA followed by Tukey	8.631	Stiff CGS vs. Stiff CGS^{Stim}	0.007
Figure S3a (*Map2*)	ANOVA followed by Tukey	21.9	Glass vs. Stiff CGS^{Stim}	0.0003
Figure S3a (*Map2*)	ANOVA followed by Tukey	21.9	Stiff CGS vs. Stiff CGS^{Stim}	0.0114
Figure S3a (*Syn1*)	ANOVA followed by Tukey	7.753	Soft CGS vs. Soft CGS^{Stim}	0.0091
Figure S3b(*Cntf*)	ANOVA followed by Tukey	15.3	Glass vs. Stiff CGS^{Stim}	0.0012
Figure S3b (*Cntf*)	ANOVA followed by Tukey	15.3	Stiff CGS vs. Stiff CGS^{Stim}	0.0086
Figure S3c (CNTF)	ANOVA followed by Tukey	12.3	Glass vs. Stiff CGS	0.3648
Figure S3c (CNTF)	ANOVA followed by Tukey	12.3	Glass vs. Stiff CGS^{Stim}	0.0024
Figure S3c (CNTF)	ANOVA followed by Tukey	12.3	Stiff CGS vs. Stiff CGS^{Stim}	0.0196
Figure S3f	ANOVA followed by Tukey	12.77	Glass vs. Soft CGS	0.0009
Figure S3f	ANOVA followed by Tukey	12.77	Glass vs. Soft CGS^{Stim}	0.0005
Figure S3f	ANOVA followed by Tukey	12.77	Stiff CGS vs. Soft CGS	0.0051
Figure S3f	ANOVA followed by Tukey	12.77	Stiff CGS vs. Soft CGS^{Stim}	0.0028
Figure S3f	ANOVA followed by Tukey	12.77	Stiff CGS^{Stim} vs. Soft CGS	0.0210
Figure S3f	ANOVA followed by Tukey	12.77	Stiff CGS^{Stim} vs. Soft CGS^{Stim}	0.0114
Figure S4b	ANOVA followed by Tukey	11.83	Negative vs. CNTF	0.0001
Figure S4b	ANOVA followed by Tukey	11.83	VEGFA vs. CNTF	0.0005
Figure S4b	ANOVA followed by Tukey	11.83	BDNF vs. CNTF	0.0044
Figure S4b	ANOVA followed by Tukey	11.83	NT3 vs. CNTF	0.0020
Figure S5b	ANOVA followed by Tukey	103.3	Control vs. Scramble^{KD}	0.9890
Figure S5b	ANOVA followed by Tukey	103.3	Control vs. CNTFK^{D}	<0.0001
Figure S5b	ANOVA followed by Tukey	103.3	Scramble^{KD} vs. CNTFK^{D}	<0.0001

Table S4. Details of statistical analysis.

Figures

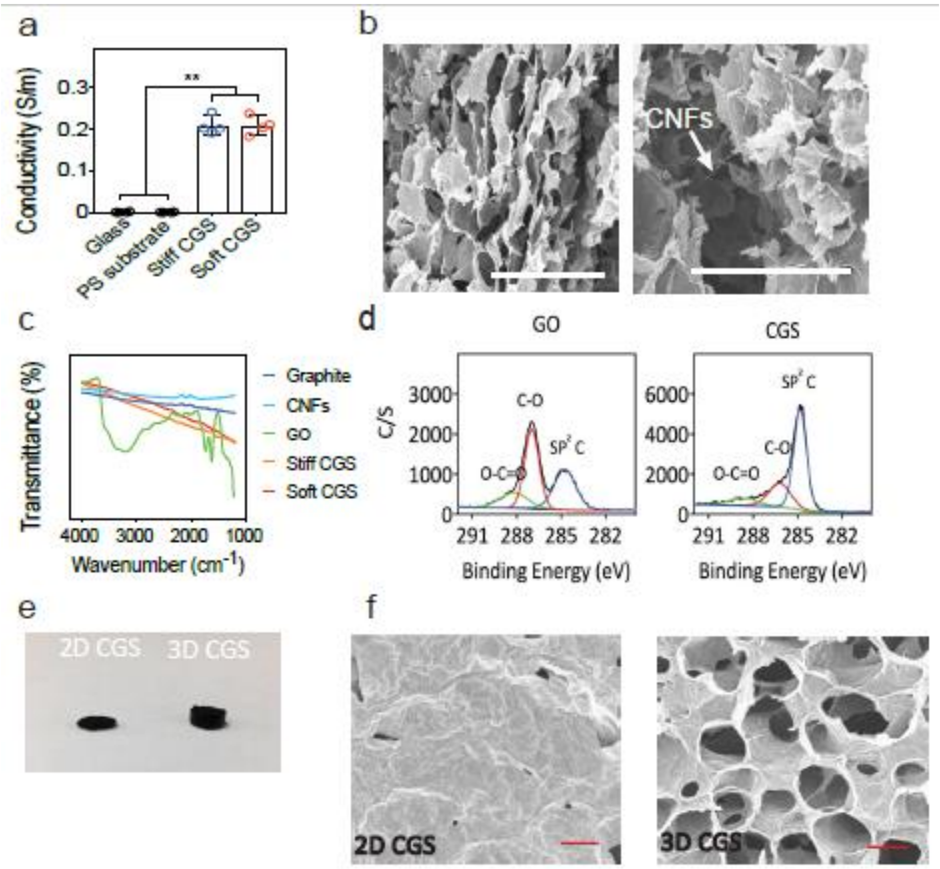

Figure S1. Characterization of CGS.

(a) Electrical conductivity of different substrates including glass, PS (polystyrene) substrate, Stiff CGS (~12 kPa), and Soft CGS (~3 kPa)). **(b)** SEM images of CGS. Scale bars indicate 100 (Left) and 50 µm (Right), respectively. **(c)** FTIR analysis of varying substrates including graphite, CNFs, GO, Stiff and Soft CGS after reducing GO by chemical reduction. FT-IR results showed that GO was fully reduced by reducing agents, forming CGS. In the presence of CNFs, CGS (Soft-CGS) showed negligible oxide groups, such as –OH (~3200 cm^{-1}) and –COOH (~1700 cm^{-1}). **(d)** XPS analysis of GO and CGS. XPS analysis also showed that GO contained oxides groups including C-O and O-C=O in histogram, whereas CGS did not have high C-O and O-C=O bands. Interestingly, CGS showed a high peak of the sp^2 carbon band, indicating that during oxide reduction, rGO was intertwined with CNFs due to a hydrophobic interaction; eventually forming the CGS. **(e)** Photograph comparing 2D CGS and 3D CGS. **(f)** SEM images comparing 2D CGS versus 3D CGS surfaces. Scale bars 40 µm.

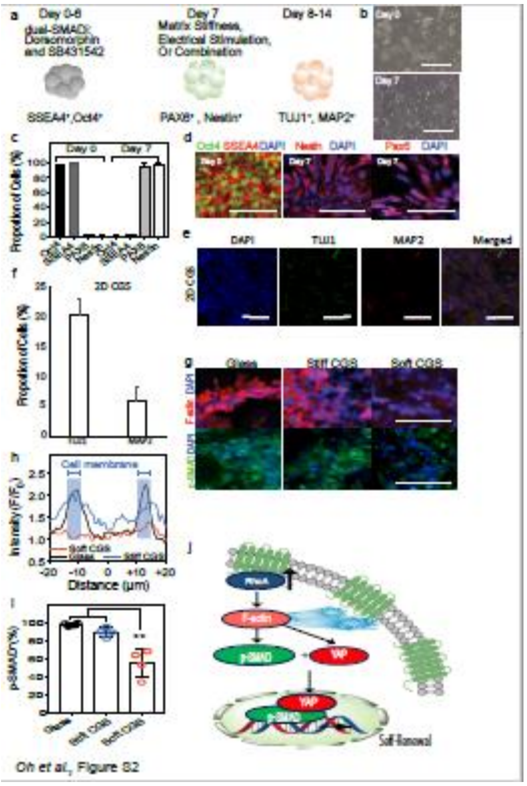

Figure S2. (a) Schematic illustration of cell culture procedure. **(b)** Contrast images of iPSCs at 0d and after 7d pre-treatment. Scale bars indicate 200 μm. **(c)** Proportion of positive cells in cell culture for 0 d and 7 d. Most iPSC markers including Oct4 and SSEA4 disappeared over 7d culture, whereas neural progenitor markers such as Nestin and PAX6 become strongly expressed. Values represent the mean of independent experiments (*n* = 4); error bars, S.D. **(d)** Representative images of iPSC cultures on 0d and 7d. Scale bars indicate 50 μm. **(e)** Representative immunocytofluorescence analysis of TUJ1 (Green) /MAP2 (Red) in iPSCs cultured on 2D CGS. Cell nuclei were counterstained with DAPI (Blue). Scale bars indicate 50 μm. **(f)** Bar plot showing the percentage of cells labeled with $TUJ1^+$ or $MAP2^+$ at 7 d on 2D CGS. Values represent the mean of independent experiments (*n* = 4); error bars, S.D. **(g)** Representative immunofluorescence images indicating that Soft CGS promotes neuronal conversion by degrading polymerization of F-actin and exclusion of p-SMAD from the nucleus. Cells were stained with F-actin (Red) and p-SMAD (Green). Polymerization of F-actin in iPSCs on the CGSs of varying elasticity. Nuclei are shown in blue. Scale bars indicate 50 μm. **(h)** Histogram showing profile of F-actin. Cells on glass and Stiff CGS (*E*=12 kPa) exhibited more intense cytoplasmic F-actin staining than cells on Soft CGS (*E*=3 kPa). **(i)** Bar plot showing rigidity-dependent subcellular localization of p-SMAD in iPSCs cultured for 2 d on different

substrates. Percentage of p-SMAD indicates that p-SMAD was co-localized with DAPI. **(i)** Analyzed using a one-way ANOVA, followed by Tukey's HSD *post hoc* test with ** $P < 0.01$. Values represent the mean of independent experiments ($n = 4$); error bars, S.D. **(j)** Schematic diagram of YAP and p-SMAD localization into cell nucleus. The stiff substrates induce RhoA expression. In this cascade, F-actin expression is accelerated by RhoA, resulting in co-localization of YAP and p-SMAD. This co-localization in the cell nucleus subsequently induces transcription factors which are mainly utilized for self-renewal and preventing the cell differentiation process.

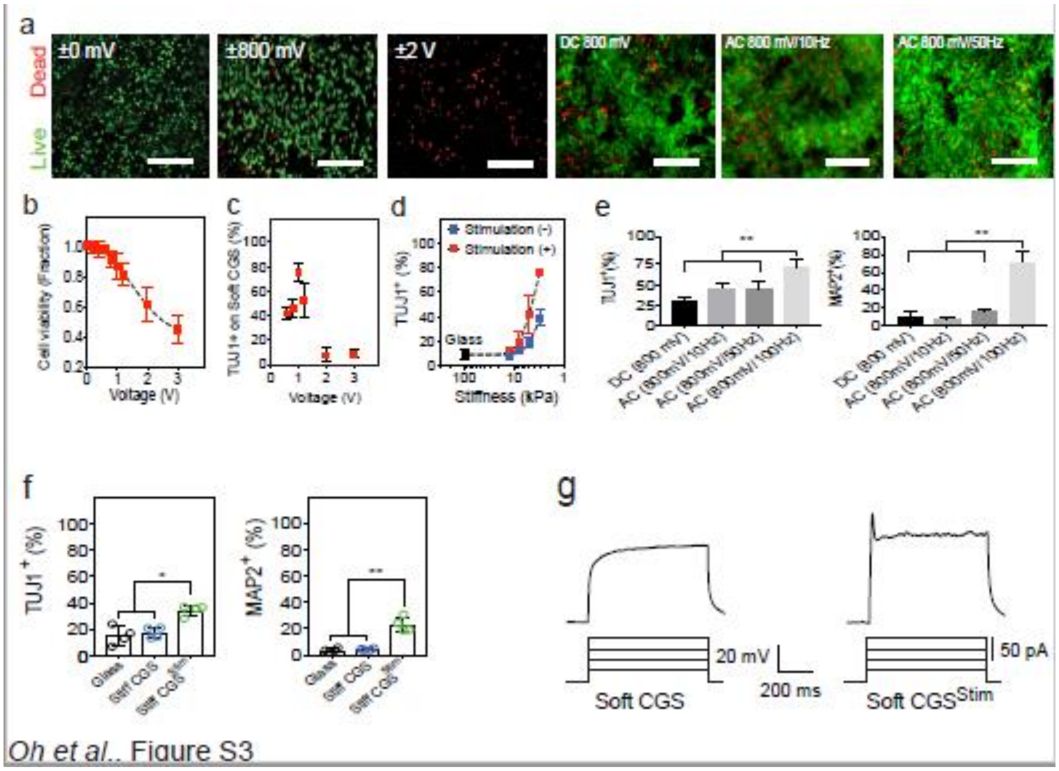

Figure S3. Optimized electrical stimulation induces efficient neuronal conversion of iPSCs on CGS.

(a) Cell viability analysis using Live/Dead staining. Frequency 100Hz unless otherwise noted in upper left hand corner. Stimulation for 1hr. Green (calcein AM) and red (ethidium homodimer-1) indicate viable and dead cells, respectively. Scale bar indicates 100 µm. **(b)** Cell viability analysis across different applied voltages ranging from 0 to ±2 V at 100Hz for 1 hr. **(c)** Proportion of $Tuj1^+$ cells on Soft CGS with varying voltages at 100Hz for 1 hr. **(d)** Proportion of $Tuj1^+$ cells with or without an exposure to the stimulation (±800 V/100 Hz for 1 hr) on different substrate stiffness. **(e)** $TUJ1^+$ and $MAP2^+$ at 7 d on CGSs of varying frequency patterns including

DC, AC 10Hz, and AC 50Hz. 800 mV was utilized to stimulate the cells. **(f)** Bar plots showing the electrical stimulation-induced increase in TUJ1 and MAP2 on Stiff CGS. Analyzed using a one-way ANOVA, followed by Tukey's HSD *post hoc* test with * and ** $P < 0.05$ and 0.01. Values represent the mean of independent experiments ($n = 4$); error bars, S.D. **(g)** The electrophysiological properties of iPSC-derived neurons after 7 days of culture demonstrated representative traces of membrane potential changes with step current injections in iPSC-derived neurons cultured 7 days on Soft CGS without (left) and with (right) one time electrical stimulation (Soft CGS^{Stim}). A single spikelet was observed only in Soft CGS^{Stim} condition. **(f)** Analyzed using a one-way ANOVA, followed by Tukey's HSD *post hoc* test with * and ** $P <$ 0.05 and 0.01. Values represent the mean of independent experiments ($n = 4$); error bars, S.D.

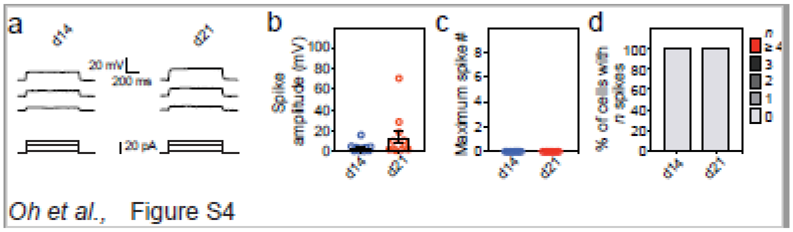

Figure S4. Standard small molecule technique doesn't show efficient immunofluorescent and electrophysiological characterization of iPSC-derived neurons.

(**a**) Representative membrane potentials upon step current injections of iPSC-derived cells cultured 14 d and 21 d on glass substrate with small molecule technique. (**b**) The summary results of averaged spike amplitude from iPSC-derived cells on 14 d and 21 d. **(c and d)** Maximum spike number and percentage of cells with indicated firing frequencies at 14 d and 21 d of differentiation on glass substrate with small molecule technique.

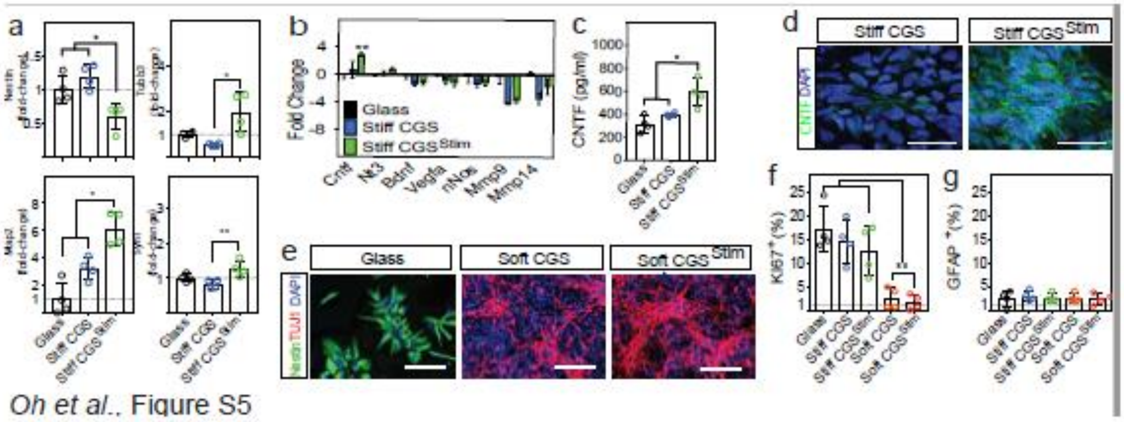

Figure S5. Electrical stimulation augments CNTF production in iPSCs cultured on Stiff CGS.

(a) qRT-PCR analysis of neuronal markers: *Nestin*, neuroectodermal stem cell marker; *Tubb3*, early neuronal marker*; Map2 and Syn1*, matured-neuronal marker at 7 d on Glass, Stiff CGS, and Stiff CGS^{Stim}. **(b)** The expression levels of neurotrophic factor genes at 1d after the electrical stimulation. Data are represented as Log_2 such that positive values indicate upregulation and negative values indicate downregulation relative to cells on glass substrate. **(c)** CNTF in the supernatants of samples with varying substrates, determined by sandwich ELISA. **(d)** Immunocytochemistry analysis for the CNTF (Green) on Stiff CGS and Stiff CGS^{Stim}. Cell nuclei are counterstained with DAPI (Blue). Scale bars indicate 25 µm. **(e)** Immunocytochemistry analysis for early neuronal differentiation (TUJ1, Red) and neural progenitors (Nestin, Green). Scale bar indicates 200 µm. Cell nuclei were counterstained with DAPI (Blue). **(f and g)** Percentages of cell proliferation marker (**f**, Ki67) and glial cell (**g**, GFAP) at 7 d on CGSs with or without an exposure to the electrical stimulation. **(a-c, f-g)** Analyzed using a one-way ANOVA, followed by Tukey's HSD *post hoc* test with * and ** $P < 0.05$ and 0.01. Values represent the mean of independent experiments ($n = 4$); error bars, S.D.

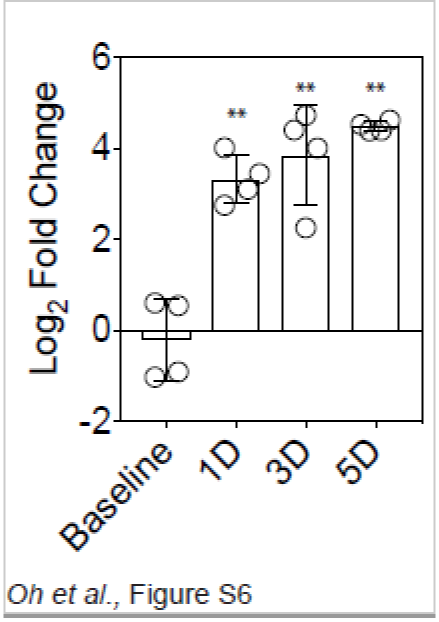

Figure S6. Quantification of the CNTF transcription at 5 days after stimulation from the cells. qRT-PCR analysis of CNTF for 5d on Soft CGS^{Stim}. Analyzed using a one-way ANOVA, followed by Tukey's HSD *post hoc* test with ** $P < 0.01$ compared to baseline. Values represent the mean of independent experiments ($n = 4$); error bars, S.D.

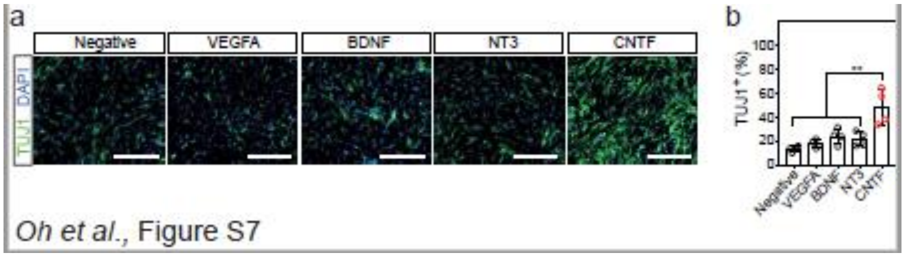

Figure S7. An exposure to CNTF promotes neuronal differentiation of iPSCs on soft CGS. **(a)** Immunocytochemistry analysis for TUJ1 of iPSCs on Glass. After preconditioning first 7 d, cells were passaged on glass and cells were maintained with N2B27 media in the presence of various neurotrophic factors including VEGFA, BDNF, NT3, and CNTF (1 ng/ml). Cell nuclei were counterstained with DAPI (Blue). Scale bar indicates 200 μm. **(b)** Quantification of TUJ1^{+} at 14 d of differentiation. **(b)** Analyzed using a one-way ANOVA, followed by Tukey's HSD *post hoc* test with ** $P < 0.01$. Values represent the mean of independent experiments ($n = 4$); error bars, S.D.

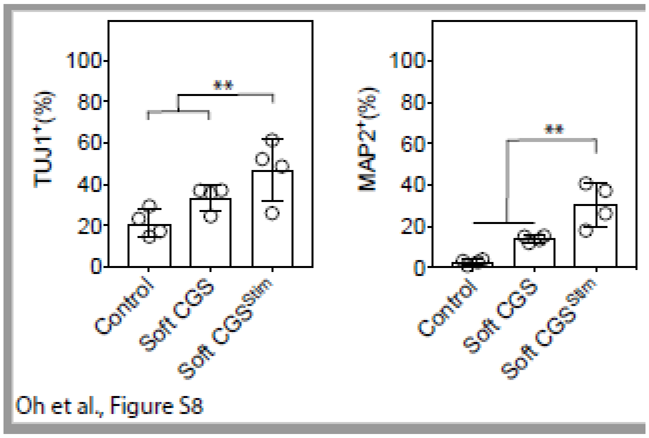

Figure S8. Assessment of immature and more mature neuronal markers (TUJ1 and MAP2) to demonstrate that combination of mechanical and electrical cues promotes earlier conversion of the iPSC-derived neurons. Bar plot showing the percentage of cells labeled with $TUJ1^+$ or $MAP2^+$ on CGS substrates. Analyzed using a one-way ANOVA, followed by Tukey's HSD *post hoc* test with ** $P < 0.01$. Values represent the mean of independent experiments (n = 4); error bars, S.D.

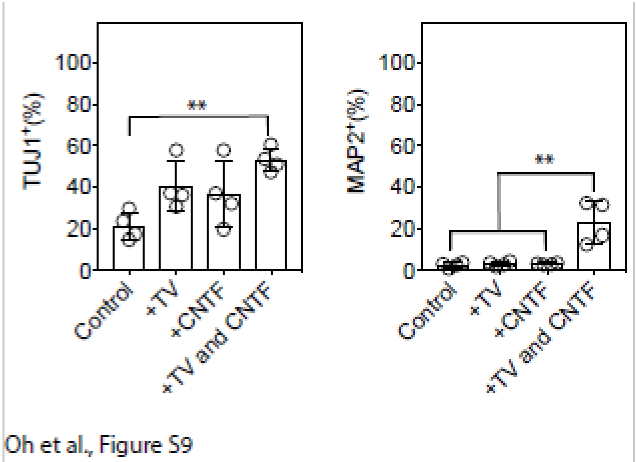

Figure S9. Assessment of immature and more mature neuronal markers (TUJ1 and MAP2) to demonstrate that addition of TV and CNTF promotes earlier conversion of the iPSC-derived neurons. Bar plot showing the percentage of cells labeled with $TUJ1^+$ or $MAP2^+$ with the addition of factors. Analyzed using a one-way ANOVA, followed by Tukey's HSD *post hoc* test with ** $P < 0.01$. Values represent the mean of independent experiments (n = 4); error bars, S.D.

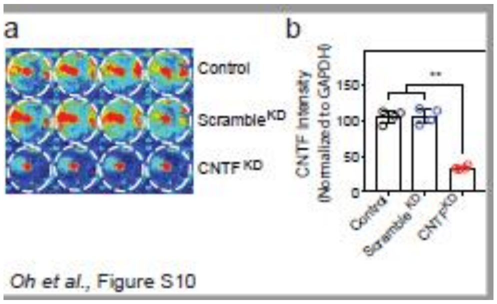

Figure S10. CNTF expression was successfully down-regulated by CNTF shRNA.

(a) Representative in-cell western images of human iPSCs in different treatment conditions such as media (Control), Scramble knock-down (Scramble^{KD}), and CNTF knock-down (CNTF^{KD}). **(b)** Quantification of in-cell western images per groups. CNTF intensity was normalized to GAPDH expression. **(b)** Analyzed using a one-way ANOVA, followed by Tukey's HSD *post hoc* test with ** $P < 0.01$. Values represent the mean of independent experiments ($n = 4$); error bars, S.D.
